# On Nb Silicide Based Alloys: Alloy Design and Selection

**DOI:** 10.3390/ma11050844

**Published:** 2018-05-18

**Authors:** Panos. Tsakiropoulos

**Affiliations:** Department of Materials Science and Engineering, The University of Sheffield, Sheffield S1 3JD, UK; p.tsakiropoulos@sheffield.ac.uk; Tel.: +44-114-222-5960

**Keywords:** intermetallics, alloy design, creep, oxidation, high temperature alloys

## Abstract

The development of Nb-silicide based alloys is frustrated by the lack of composition-process-microstructure-property data for the new alloys, and by the shortage of and/or disagreement between thermodynamic data for key binary and ternary systems that are essential for designing (selecting) alloys to meet property goals. Recent publications have discussed the importance of the parameters δ (related to atomic size), Δχ (related to electronegativity) and valence electron concentration (VEC) (number of valence electrons per atom filled into the valence band) for the alloying behavior of Nb-silicide based alloys (J Alloys Compd 748 (2018) 569), their solid solutions (J Alloys Compd 708 (2017) 961), the tetragonal Nb_5_Si_3_ (Materials 11 (2018) 69), and hexagonal C14-NbCr_2_ and cubic A15-Nb_3_X phases (Materials 11 (2018) 395) and eutectics with Nb_ss_ and Nb_5_Si_3_ (Materials 11 (2018) 592). The parameter values were calculated using actual compositions for alloys, their phases and eutectics. This paper is about the relationships that exist between the alloy parameters δ, Δχ and VEC, and creep rate and isothermal oxidation (weight gain) and the concentrations of solute elements in the alloys. Different approaches to alloy design (selection) that use property goals and these relationships for Nb-silicide based alloys are discussed and examples of selected alloy compositions and their predicted properties are given. The alloy design methodology, which has been called NICE (Niobium Intermetallic Composite Elaboration), enables one to design (select) new alloys and to predict their creep and oxidation properties and the macrosegregation of Si in cast alloys.

## 1. Introduction

Performance targets of future aero-engines have been revised to meet significant reductions in harmful emissions. For example, ACARE (Advisory Council for Aircraft Innovation and Research in Europe) used data for the year 2000 as reference to set goals to be met by aviation by the year 2050, also known as FLIGHTPATH 2050. These require reductions (a) of aircraft CO_2_ emissions by 75% per passenger kilometer, (b) of aircraft noise by 65% and (c) of NO_x_ (oxides of nitrogen) emissions by 90%. The engine contribution to these goals is reduction of CO_2_ by 30% and of the NO_x_ certification metric by 75%.

These goals could be met if there were materials available that would enable the engines to operate with significantly higher turbine entry temperatures in the range 1850 °C to 1900 °C. Thus, new materials with capabilities beyond those of Ni based superalloys are required. The need for such materials and the property goals that must be met by them have been well documented in the literature [1]. For example, the creep goal for Nb-silicide based alloys is as follows: “the creep strength should be greater than 170 MPa at a creep rate of 2·10^−8^ s^−1^ at 1200 °C” [1]. The creep goal assumes density of Nb-silicide based materials of 7 g/cm^3^. The search for new materials has considered alloys and composites that utilize the attractive high temperature properties of refractory metal intermetallics [2]. Alloys and in situ composites exploiting the properties of refractory metal silicides have the potential to offer a balance of properties to meet performance targets. Nb-silicide based alloys (or Nb-silicide in situ composites) and Mo-Si-B based alloys belong in this category of new materials. This paper is about the design (selection) of Nb-silicide based alloys. 

Alloy design requires a set of properties that the new alloy must have to satisfy performance targets. Data about the latter and the material property goals has been provided by regulatory authorities and the relevant industries (see above and [1]). The property goals are expected to guide the development of new alloys for ultra-high temperature structural applications beyond those for the Ni based superalloys. Alloy design also requires data that links (i) a particular set of properties with specific microstructure(s) and (ii) a particular process with microstructure, in other words, alloy designers need composition-process-microstructure-property relationships, which usually give a good (reasonable) description of particular phenomena over a restricted range of parameters. Armed with the aforementioned data and knowledge about parameters that control material properties, the alloy developer can design (select) alloy compositions using thermodynamic data from databases and/or derived using CALPHAD (CALculation of PHAse Diagrams) and/or ab initio calculations and other alloy design tools, e.g., PHACOMP (PHAseCOMPutation), neural networks [3,4]. Equilibrium calculations will specify the ultimate microstructure, for example give the volume fractions of phases as function of alloy composition and temperature. However, the results of such calculations are far from the actual microstructures that exist in the alloy under anticipated service conditions.

Research on ferrous and non-ferrous (i.e., Al, Cu, Mg, Ni, Ti) alloys, which has been on-going for many decades, has provided large volume of experimental data that has helped to calibrate alloy design tools and improve their accuracy and reliability. However, even with all the available data for the aforementioned alloys, (a) phase diagrams are reliable only in those areas for which there is experimental data, (b) different thermodynamic databases can disagree for the same material type, (c) the CALPHAD method is most reliable when interpolating between compositions used to build the database and (d) models about mechanical behavior are suitable only for specific alloys of an alloy family.

What is the current situation with Nb-silicide based alloys? The important phases in their microstructures are the bcc Nb solid solution (Nb_ss_) and tetragonal 5-3 Nb silicide Nb_5_Si_3_. The latter exists in two forms, both of which have the same crystal structure but different lattice parameters, namely the high temperature βNb_5_Si_3_ (tP32 D8_m_, prototype W_5_Si_3_) and the low temperature αNb_5_Si_3_ (tP32 D8_l_, prototype Cr_5_B_3_). The Nb_5_Si_3_ silicide also can form as metastable hexagonal γNb_5_Si_3_ (hP16, D8_8_, prototype Mn_5_Si_3_). The βNb_5_Si_3_ can form in situ either as a primary phase or via the metastable eutectic reaction L → Nb + βNb_5_Si_3_, the αNb_5_Si_3_ can form from the peritectoid Nb_3_Si + βNb_5_Si_3_ → αNb_5_Si_3_ and eutectoid βNb_5_Si_3_ → αNb_5_Si_3_ + NbSi_2_ transformations or from the eutectoid transformation Nb_3_Si → Nb + αNb_5_Si_3_ and the Nb_3_Si can form from the eutectic reaction L → Nb + Nb_3_Si [1,2]. The aforementioned eutectics can be grown using directional solidification (DS).

The Nb_ss_ and tetragonal Nb_5_Si_3_ are desirable phases respectively for fracture toughness and creep. Nb alloys and Nb intermetallics can oxidize catastrophically (pest oxidation) at temperatures between 600 °C and 900 °C. The Nb_ss_ is the Achilles’ heel for the oxidation of Nb-silicide based alloys. Alloying of both phases is essential to meet the oxidation property goal. However, such alloying for oxidation resistance can have adverse effect on the toughness and creep properties of the phases and alloys. In other words, there is a competition between toughness and creep against oxidation resistance. Other intermetallics and other solid solutions also can be stabilized in the microstructure of Nb-silicide based alloys [5,6,7]. Depending on alloying additions and their concentrations in the alloy and on processing conditions, different types of Nb_ss_ (see [6]) and tetragonal Nb_3_Si, C14-AB_2_ Laves phase(s) (mainly NbCr_2_ based) and A15 Nb_3_X (X = Al, Ge, Si, Sn) intermetallics can form [7,8]. Nb-silicide based alloys with AB_2_ Laves and A15 phases in their microstructures exhibit better oxidation. However, the Laves and A15 phases can have a negative effect on the toughness and creep of the alloys. Their volume fraction(s) must be optimized to achieve a balance of properties.

The most recent research on Nb-silicide based alloys has mainly focused on “forth” generation alloys, which have evolved from Nb-Ti-Si-Al-Cr alloys [9], MASC (Metal And Silicide Composite) type [1] Nb-Ti-Si-Al-Cr-Hf-(Sn, Ge) alloys, Nb-Si-TM-RM alloys to Nb-Si-TM-RM-X alloys (TM = Cr, Hf, Ti, Zr, RM = Mo, Ta, V, W, and X = Al, B, Ge, Sn, and exotic addition(s) [5,6,7,8,9,10,11,12]). To date, almost all the alloys have been studied in their cast and heat-treated conditions. There is very little research on simple ternary or quaternary alloys that were cast, and then extruded or forged and/or HIPed (Hot Isostatic Pressed) and heat treated, i.e., on the down processing of alloys, and even less research on simple ternary or quaternary powder metallurgy (PM) alloys. Some Nb-silicide based alloys are very close to or even surpass specific property goals.

The volume of experimental data for Nb-silicide based alloys is miniscule compared with that for ferrous and non-ferrous alloys. The alloys tend to have very high liquidus temperatures, in excess of 2000 °C. For research purposes the alloys are made as small buttons (in most cases weight ≤ 20 g) using non-consumable (W) electrode arc melting or plasma melting. Bars with ≤ 10 mm diameter also have been grown using optical float zone (OFZ) melting. Severe macrosegregation of Si and other alloying elements is present in many cast alloys [13] and often the chemical inhomogeneity prevails after heat treatment(s). Toughness or compressive creep has been measured using specimens cut from cast buttons or OFZ bars and the data for tensile creep is limited for few simple alloys. Oxidation behavior below and above 1000 °C has been studied mainly using isothermal oxidation (e.g., [14,15]). There are very few studies of cyclic oxidation behavior. Toughness, creep and oxidation rarely have been reported for the same alloy. Composition-process-microstructure-property relationships are not available for Nb-silicide based alloys. Toughness and creep have been studied using models for composites, for example for creep see [16,17]. 

The situation is not better regarding thermodynamic and phase equilibria data for Nb-silicide based alloys. Limited experimental data is available for some thermodynamic properties [18]. Phase diagrams for RM-RM (RM = refractory metal) systems have data only above 2400 °C [19]. There are disagreements about the composition and temperature of the equilibrium eutectic reaction L → Nb + Nb_3_Si, for which reported values respectively are in the range 15.3 at.% Si [20] to 18.7 at.% Si [21] for the composition of liquid, and 1912 °C [22] to 1938 °C [23] for the eutectic temperature. Calculated Nb-Si binary phase diagrams often ignore the experimentally established solubility range of the Nb_5_Si_3_. There are also disagreements about the temperature of the eutectoid reaction Nb_3_Si → Nb + αNb_5_Si_3_, which is reported to be in the range 1666 °C [23] to 1770 °C [19]. The temperature of the Ti_5_Si_3_ + βTi → Ti_3_Si peritectoid reaction is considered to be 1170 °C, based on [24], but there is new data that shows that it is in the range 1225 °C < T < 1250 °C [25].

There are also disagreements between experimental and calculated liquidus projections of the Nb-Ti-Si ternary system. For example, experimental [26] and calculated [20] liquidus projections do not specify the type of Nb_5_Si_3_ (β or α) forming from the melt, the calculated projections in [27,28] disagree with that in [29] and the liquidus projections in [28] and [30] differ significantly. In other words, there is disagreement about the effect Ti has on the stability of alloyed tetragonal (Nb,Ti)_5_Si_3_. The situation is also not better for the Nb-Cr-Si ternary system, for which there is disagreement about the liquidus projection [31,32] and three phase Nb_ss_-Nb_5_Si_3_-NbCr_2_ equilibrium at 1500 °C and lower temperatures [33,34,35]. Furthermore, there is no data for important ternary systems of Nb with Sn or Ge (both elements improve significantly oxidation resistance), namely the Nb-Sn-X (X = Al, Hf, Mo, Ta, Ti, V, W), Nb-Ge-Z (Z = Cr, Mo, Ta, W), and Nb-Si-Ta systems [36]. In their review of Laves phases, Stein et al. [37] concluded that there are also “significant problems associated with the experimental determination of phase equilibria involving Laves phases”.

Titanium improves the oxidation of Nb [38] and Nb-silicide based alloys [15]. The solubility of Ti in the Nb_ss_ can vary significantly depending on alloying additions [9,39]. The actual concentration of specific solutes in Nb_ss_ depends on the concentration of Ti in the solid solution [40]. For example, the concentration of Cr in the Nb_ss_ increases with that of Ti. Furthermore, the partitioning of TM (=transition metal) and RM elements in the Nb_ss_ can result to strong solid solution strengthening of the Nb_ss_ and can affect the Si concentration in the Nb_ss_ [6]. In Nb-silicide based alloys it is possible to have three types of bcc solid solution, namely “normal” Nb_ss_, Ti rich Nb_ss_ and Nb_ss_ with no Si [6]. In the alloyed Nb_5_Si_3_ the Nb can be substituted by other transition and refractory metals and the Si by other simple metals and metalloid elements. An actual 5-3 silicide composition determined by electron probe microanalysis (EPMA) is [48.3Nb-7.7Ti-1.8W-0.9Hf-0.8V-0.3Cr]-(33.1Si-3.2Ge-2.8Al-1.1Sn). Examples of actual compositions of other intermetallics are [26.4Nb-12Ti-5.8Mo-2.5Hf-0.9W]-(35.8Cr-8.2Si-5.5Al-1.6Sn-1.3Ge) for a Laves phase and [51.2Nb-28.1Ti-2.1Cr-1Fe-0.7Hf]-(3.8Si-2.7Al-10.4Sn) for an A15 phase, also determined by EPMA. In the compositions of the latter phases the elements substituting Nb are in the square brackets and those substituting Si in the parentheses. Modelling such real phases in CALPHAD or ab initio calculations is not a simple matter. For example, in the crystal structure of αNb_5_Si_3_ there are four different sub-lattices. 

Alloying element additions reported in Nb-silicide based alloys include Al, B, Cr, Fe, Ga, Ge, Hf, Ho, Mo, Si, Sn, Ta, Ti, V, W, Y, Zr. Some of the alloying additions provide solid solution strengthening to the Nb_ss_ (for example, Mo, Ta, Ti, W), other elements suppress pest oxidation and improve oxidation at high temperatures (for example, Al, B, Cr, Fe, Ge, Hf, Sn, Ti), other elements suppress the stable eutectic and replace it by the metastable one (for example, Al, Mo, Sn, Ta, W) and other elements stabilize tetragonal Nb_5_Si_3_ (for example, Al, Cr, Mo, Ta, W) and improve creep (Mo, Ta, W). Are all these alloying elements essential additions in Nb-silicide based alloys to meet a property goal? How can the alloy designer select an alloy to get a balance of properties? How sensitive are the alloys to deviations from desirable (ideal) compositions? Can alloys containing large concentrations of very expensive elements be justified? Which are the compositional freedoms for primary alloying constituents? What are the maximum tolerances for minor alloying additions? The latter two questions were considered in [41] for eutectics with Nb_ss_ and Nb_5_Si_3_ that form in Nb-silicide based alloys. 

The above brief discussion of the status quo for Nb-silicide based alloys shows that the tools available to the alloy designer are very limited compared with what is available for the development of ferrous and non-ferrous alloys. The motivation for the research presented in this paper was to attempt to provide answers to the above questions and to find out whether existing data for Nb-silicide based alloys can lead us to a complementary alloy design (selection) route that can assist alloy development when used with other alloy design tools that are improved continuously as on-going research generates much needed experimental thermodynamic data and phase equilibria data for key ternary Nb based systems [42,43,44]. 

How can one study alloying behavior in Nb-silicide based alloys? Actual compositions (at.%) of Nb with no Si solid solutions observed in Nb-silicide based alloys are 21.3Nb-11.3Ti-23.4Mo-18.0W-17.7Cr-5.3Al-3.0Sn, 25.8Nb-6.0Ti-21.8Mo-29.4W-12.0Cr-5.0Al and 15.4Nb-11.9Ti-27.1Mo-24.0W-13.3Cr-5.2Al-3.1Sn. Actual compositions (at.%) of Nb-silicide based alloys are 25Nb-27.4Ti-11.8Si-8.9Sn-7.2Mo-6.8Al-6.6Cr-6.3Ge, 27.0Nb-27.0Ti-14.0Si-8.5Sn-6.7Mo-7.0Ge-4.8Cr-5.0Al and 35.0Nb-17.0Ti-15.8Si-5.0Mo-5.0W-6.0Sn-5.5Cr-5.4Al-5.3Ge. Actual compositions (at.%) of eutectics with Nb_ss_ and Nb_5_Si_3_ observed in Nb-silicide based alloys are 38.8Nb-30.6Ti-13.4Si-7.3Hf-5.4Al-4.4Sn and 34.3Nb-36.8Ti-21Si-7.9Hf. All the above compositions were determined using electron probe microanalysis (EPMA). They satisfy the “standard definition” of the so called “high-entropy alloys” (HEAs), “concentrated solid solution alloys” (CSSAs), “multi-principle element alloys” (MPEAs), “complex concentrated alloys” (CCAs) [45] (note that it is not suggested that all Nb-silicide based alloys are HEAs). Amorphous Nb-Si alloys can be produced by Rapid Solidification techniques. Enthalpy and entropy of mixing, atomic size, electronegativity and valence electron concentration have been considered for the study of the alloying behavior of crystalline and amorphous alloys. Parameters used for the study of HEAs etc include the atomic size difference (δ), electronegativity difference (Δχ), valence electron concentration (VEC), entropy (ΔS_mix_) and enthalpy (ΔH_mix_) of mixing and Ω = T_m_ ΔS_mix_/|ΔH_mix_| [45].

The research presented in this paper builds on earlier studies of the alloying behavior of Nb-silicide based alloys [46], their solid solutions [6], the tetragonal Nb_5_Si_3_ [47], and hexagonal C14-NbCr_2_ and cubic A15-Nb_3_X phases [48] and eutectics with Nb_ss_ and Nb_5_Si_3_ [41]. For the Nb solid solutions formed in Nb-silicide based alloys it was shown that the parameters δ, Δχ, VEC, ΔS_mix_, ΔH_mix_ and Ω could describe the alloying behavior (the capital letter Q was used instead of Ω for the ratio T_m_ΔS_mix_/|ΔH_mix_| in [6] to avoid confusion with the term Ω_ij_ in the definition of ΔH_mix_). The parameters δ and Δχ respectively separated Ti rich Nb_ss_ and Nb_ss_ with no Si, and solid solutions depending on alloying additions.

The alloys, where the solid solutions studied in [6] belonged to, were studied in [46] using the same parameters and the ratio sd/sp of sd electronic configuration elements over sp elements. It was shown that the alloys could be separated in three groups according to alloying additions using the parameters Δχ, VEC, and δ. When the data for the parameters of alloys and solid solutions were combined it was discovered that specific pairs of parameters could separate the alloys and their bcc solid solutions. This research also revealed that there is an overlap of the values of some parameters when Nb-silicide based alloys are compared using data for best isothermal oxidation at 800 °C and 1200 °C and for creep at 1200 °C and 210 MPa.

The alloying behavior and properties, respectively of tetragonal Nb_5_Si_3_, and hexagonal C14-NbCr_2_ and cubic A15-Nb_3_X phases, which were in the Nb-silicide based alloys studied in [46], were studied respectively in [47,48]. The alloying of Nb_5_Si_3_ was described using Δχ versus VEC maps. Deterioration of the creep of alloyed Nb_5_Si_3_ was accompanied by decrease of VEC and increase or decrease of Δχ depending on alloying addition(s). A plot of Δχ versus Cr, and maps of Δχ versus VEC and VEC versus atomic size separated the alloying behavior of C14-NbCr_2_. The better creep of Nb(Cr,Si)_2_ compared with the unalloyed Laves phase was related to the decrease of the parameters VEC and Δχ. The Δχ versus VEC map separated the alloying behavior of elements in A15-Nb_3_X, the hardness of which was correlated with the parameters Δχ and VEC.

The aims of the research presented in this paper were (i) to find out relationships between the alloy parameters Δχ, VEC, δ and solute concentrations in Nb-silicide based alloys and the oxidation and creep properties of these alloys and (ii) to demonstrate the use of such relationships to design (select) new Nb-silicide based alloys. The toughness of Nb-silicide based alloys is not addressed in this paper. The structure of the paper is as follows. First the objectives of the research are given. Then oxidation and creep of Nb and Nb-silicide based alloys are discussed to highlight the importance of aforementioned parameters for the study of these properties. Relationships between parameters and oxidation, creep or Si macrosegregation are shown and finally a methodology for designing (selecting) new Nb-silicide based alloys is presented.

## 2. Objectives, Results and Discussion

The first objective of the research was to justify the use of the parameters δ, Δχ and VEC for the study of the oxidation and creep of Nb-silicide based alloys. The second objective was to locate the phases that can form in Nb-silicide based alloys in a Δχ versus VEC map and to compare their creep. The third objective was to find out relationships between the alloy parameters Δχ, VEC, δ and weight gain in isothermal oxidation and steady state creep rate of Nb-silicide based alloys. These objectives were realistic because there was data for the actual compositions of the alloys for which oxidation (weight gain) or compressive creep data were available and thus it was possible to calculate the alloy parameters Δχ, VEC and δ as described in [6]. The forth objective was to find out if the aforementioned relationships can lead to the development of an alloy design/selection methodology.

Grouping of solutes in Nb-silicide based alloys was demonstrated in [6,46]. In the latter paper, correlations between activation energy for diffusion, diffusivity, atomic size and electronegativity were discussed. The research described in this paper was also interested to find out if there are correlations between atomic size, electronegativity or VEC and (i) shear moduli of cubic symmetry alloying elements added in Nb-silicide based alloys, (ii) ratios of shear and bulk moduli for cubic and hexagonal symmetry alloying elements added in Nb-silicide based alloys, (iii) anisotropy parameters for cubic and hexagonal symmetry alloying additions, 5-3 silicides and A15 intermetallics, (iv) G/B (G is the shear modulus and B is the bulk modulus) ratios of 5-3 silicides and A15 intermetallic phases (G is the shear modulus and B is the bulk modulus) and (v) the Young’s moduli of elasticity of alloying elements in Nb-silicide based alloys. All actual compositions of alloys and phases in their microstructures that were used to calculate the parameters δ, Δχ and VEC were determined using EPMA [6,41,46,47,48]. No new experimental data were created during the course of this study.

This section starts by reflecting on the importance of atomic size, electronegativity and VEC (a) in the solubility of oxygen in Nb, the type(s) and structure(s) of the oxides formed in the scales and their importance in the oxidation of Nb-silicide based alloys and (b) in the creep of polycrystalline Nb-silicide based alloys (Nb-silicide in situ composites). The latter builds on the link between alloying of Nb_5_Si_3_, C14-NbCr_2_ and A15-Nb_3_X phases and their properties that were studied in [47,48]. The alloying behavior and properties of eutectics with Nb_ss_ and Nb_5_Si_3_ were discussed in [41].

### 2.1. Oxidation

Niobium is a group 5 transition element in the periodic table. It has high solubility for oxygen, about 9 at.% at 1950 °C [19]. Alloying aims to reduce this and to slow down the diffusion of oxygen. At 800 °C and 1200 °C the diffusion distances of oxygen in Nb after 100 h are about 2.75 and 15.8 mm, respectively [49]. Titanium reduces the diffusivity of oxygen in Nb, for example the diffusivity of oxygen in Nb-25Ti is 1/20 that in pure Nb [50]. The effect of alloying on oxygen transport kinetics in pure Nb and Nb-34Hf-21Al (at.%) at 1300 °C was demonstrated in [51].

The other two transition metals in group 5, namely V and Ta, also have high solubilities for oxygen, which respectively are about 15 at.% and 6 at.% but the elements in group 6 (Cr, Mo and W) have significantly lower oxygen solubilities [19]. The aforementioned elements can be in solution in bcc Nb together with simple metals and metalloids. Also, they substitute Nb in Nb_5_Si_3_. An example of Nb_ss_ with no Si is the solid solution 63.4Nb-11.7Ti-2.3Hf-11.1Mo-5.7W-6.1Al (see introduction and [6] for more compositions of Nb_ss_ in Nb-silicide based alloys). For an example of an actual chemical composition of a Nb_5_Si_3_ silicide see introduction.

Atomic size is important in diffusion in alloys. The diffusion of solutes to the substrate/oxide interface is one of the factors that control oxidation. Wagner showed that the oxidation rate of Ni-Pt alloys at 850 °C and 1100 °C was essentially determined by the diffusion of Ni to the alloy-NiO interface [52]. The low oxidation rate of Ni alloys with Be or Si additions compared with the high oxidation rates of Ni alloys with Mo or Cu additions was attributed to differences in the atomic size of solute and solvent [52]. Silicon and Be, and Cu and Mo respectively have smaller and larger atomic size than Ni. The different oxidation behavior was attributed to enhanced diffusion of Ni due to lattice distortion that increased oxidation rate [52]. The relationship between solute atomic size and diffusivity in Nb was discussed in [6]. 

Research has linked oxygen solubility in binary Nb alloys with the electron per atom ratio (*e/α*) [53]. The latter is often used to discuss phase stability. Minimum oxygen solubility in Nb-X (X = Mo, Re) alloys was reported for (*e/α*) ratios of 5.7 and 5.75 for Mo and Re respectively, and was suggested that minimum oxygen solubility occurs when (*e/α*) = 5.7 [54]. The latter was disputed in [55]. The author’s research group has shown that in Nb-silicide based alloys the concentration of Mo in the alloy is very important for the oxidation behavior [15].

Oxygen dissolves interstitially in octahedral holes in bcc Nb. The different solubilities of oxygen in metals arise from differences in the binding energy of an oxygen atom to an interstitial site. An oxygen atom in the lattice of an element will cause electron redistribution. As an oxygen atom creates a charge density hole, electrons are excluded from the latter and then some of the excluded electrons go back to the hole. The electrons that were displaced from the excluded region will be accommodated in unoccupied metal orbitals and thus the Fermi level will change.

Phase stability can be considered in terms of (*e/α*) (averaged valence of alloying elements in an alloy) and VEC (number of valence electrons per atom filled into the valence band). The (*e/α*) ratio is the parameter in the Hume–Rothery rules [53] and the VEC is essential to determining the Fermi level in the valence band [56]. The (*e/α*) ratio is difficult to use as a universal parameter in alloy design because its value cannot be uniquely assigned to a transition metal as it depends on the surrounding environment. Instead, VEC is a more important parameter in transition metal alloys [56].

Electronegativity (χ) describes the tendency of an atom to attract electrons. The activity of a metal is correlated with its electronegativity, and metals are categorized as very electropositive (χ < 1.4), electropositive (1.4 < χ < 1.9) and electronegative (1.9 < χ < 2.54). Very electropositive metals oxidize easily. Electropositive metals can form protective metal oxide and their surfaces tarnish in oxygen. Chromium, Ge, Sn, Ti, V belong in this category. Electronegative metals do not form very stable oxides and the latter can decompose on heating. Niobium, Mo, Ta, W belong in this category. Electropositive and electronegative elements are present in the three types of bcc Nb_ss_ [6], in the Nb_5_Si_3_ silicide, where they substitute Nb or Si [47], in the C14-NbCr_2_ Laves phase where they substitute Nb or Si and in A15-Nb_3_X compounds [48], see examples of compositions given in the introduction. 

The oxidation of Nb-silicide based alloys starts with the oxidation of Nb_ss_ grains in the surface, this is followed by the oxidation of Nb_5_Si_3_ grains (and other intermetallics) in the surface. The oxidation promotes the surface segregation of Nb that subsequently oxidizes [15,57]. This is followed by oxidation of Nb_ss_ and Nb_5_Si_3_ below the surface and even in the bulk [15] as well as of other intermetallic phases such as the C14-NbCr_2_ Laves and A15-Nb_3_X phases (this is often referred to as (phase) contamination by oxygen). The oxidation of the solid solution is more sever compared with the silicide(s) and other intermetallics [14,15,57]. The oxidation of all phases depends on their chemical composition and on the alloy microstructure (volume fractions of phases, shape, size and spatial distributions of phases) that results from processing. The oxidation includes the dissolution of oxygen and the formation of sub-oxides and of several crystalline oxides in various oxidation states and leads to a mixture of oxides or oxygen-saturated metal + oxides [14,15]. 

It is important to consider the types and structure(s) of the oxides forming the scales on Nb-silicide based alloys. Oxides in which Al, Cr, Hf, Mo, Nb, Si, Ta, Ti, V or W can participate must be considered because these elements are key for achieving a balance of properties in Nb-silicide based alloys. Not all these elements are simultaneously or necessarily present in every alloy. However, Si and Ti are always present in the alloys, both tend to segregate to Nb_ss_ grains in the surface and the segregation of Ti is more severe [58,59]. The latter was suggested to promote the selective oxidation of Ti and the early formation of TiO_2_ in the scale [58].

The oxide scales formed on Nb-silicide based alloys contain TiO_2_ (rutile), SiO_2_, Nb_2_O_5_ and niobates [14,15]. In the latter, TM, RM and simple metals can be present [14,15]. The early formation of rutile and Nb oxides in the scale of Nb-silicide based alloys is critical because many of the mixed oxides that form have structures that show analogy with the rutile structure. This is briefly discussed below.

Nb can form the oxides NbO, NbO_2_ and Nb_2_O_5_ [19], where the valence of Nb respectively is Nb^2+^, Nb^3+^ and Nb^5+^. The latter oxide is the most stable. The most commonly encountered polymorph of the Nb_2_O_5_ is the monoclinic H-Nb_2_O_5_, which is the stable form in air above 900 °C. The stoichiometry of all polymorphs is maintained by NbO_6_ octahedra. In stoichiometric and slightly reduced Nb_2_O_5_, the diffusion coefficients for oxygen are up to 200 times greater parallel to the b axis than perpendicular to this direction [60].

Rutile forms a series of “shear-type” Ti_n_O_2n−1_ structures (Magneli phases) with 4 ≤ n ≤ 10 [61]. The structures are made up of layers of rutile structure with a width of n TiO_6_ octahedra. The TiO_6_ octahedra share faces across the discontinuity or crystallographic shear plane.

Binary (mixed) oxides of Nb and solute elements like Al, or TM or RM can form. Examples include the Al, Cr and V niobates AlNbO_4_, CrNbO_4_ and VNbO_4_ and the binary (mixed) oxides of Nb_2_O_5_ with TiO_2_, Ta_2_O_5_, V_2_O_5_, MoO_3_ and WO_3_. Rutile can also form binary oxides with HfO_2_ [62] and Ta_2_O_5_ [63]. There are similarities in the structures of such binary oxides that are important for the diffusion of oxygen and solute elements. Up to about 5 mol % hafnia can be in solution in rutile [62]. 

The AlNbO_4_ niobate crystallizes in a monoclinic system and in its crystal structure blocks of ReO_3_ type can be distinguished, built from distorted MO_6_ octahedra. The CrNbO_4_ niobate has tetragonal structure (tP6) with prototype TiO_2_ (rutile) and its structure consists of (Cr,Nb)O_6_ octahedra. The VNbO_4_ also has tetragonal structure (tP6) with prototype TiO_2_ (rutile). Adherence of CrNbO_4_ scale (with small amount of Cr_2_O_3_) on NbCr_2_ Laves phase has been reported after cyclic oxidation in air at 1200 °C that gave a rate of metal loss of 4.9 µm/h [64].

A very wide range of Ti_(1−x)_Nb_x_O_2_ compositions have the ideal rutile structure [65]. Binary oxides between Nb_2_O_5_ and TiO_2_ include TiO_2_-Nb_2_O_5_ (TiNb_2_O_7_) [66], TiO_2_-3Nb_2_O_5_ (Ti_2_Nb_10_O_29_) [66,67,68], and TiNb_24_O_62_. Disorder is created by Nb^5+^ as a donor dopant in TiO_2_. The crystal structure of TiNb_2_O_7_ contains ReO_3_ blocks of corner-sharing MO_6_ octahedra. The different stoichiometries and structures of the above oxides are a consequence of variations in the size of these ReO_3_ blocks and the way they are joined together.

The metal positions in the structures are occupied by one or other of these elements in a random manner. Each metal atom (Ti and Nb) is coordinated to six oxygen atoms [69,70] forming an octahedral grouping (TiO_6_ and NbO_6_). In TiNb_2_O_7_ these blocks contain MO_6_ octahedra and form a linear column along the b-axis of the unit cell. Perpendicular to the b-axis the columns are bound by crystallographic shear planes. Across the shear planes the MO_6_ octahedra share edges [70]. In all cases, the b axis (the short axis of the structures) is around 3.8 Å, the length of the octahedral body diagonal of the structures. The smaller and lower-charged Ti^4+^ ion has a preference for the octahedra at the corners and edges of the blocks. TiNb_2_O_7_ is very friable, whereas the TiNb_2_O_6.42_ is very resistant to fracture. These properties are reversible as the binary oxide composition can change in the oxidizing atmosphere.

The structures of binary oxides in the Nb_2_O_5_-WO_3_ system [70] also derive from blocks of the ReO_3_ type, with networks of octahedral MO_6_ groups linked by sharing the oxygen atoms at their vertices. The growth axes of the Nb-W oxides are parallel to the infinite extension of the blocks, with a periodicity of 3.8 Å. The structure of Nb_14_Mo_3_O_44_ and Nb_12_MoO_33_ (isostructural oxides respectively with Nb_14_W_3_O_44_ and Nb_12_WO_33_) also consists of blocks of ReO_3_ type, built up from deformed NbO_6_ octahedra sharing only corners. Oxides in the V_2_O_5_–Nb_2_O_5_ system consist of corner sharing metal-oxygen polyhedra. The structure of VNb_9_O_25_ consists of block structures of NbO_6_ octahedra, which are shared with VO_4_ tetrahedra at each corner of those blocks [71].

The columbite-tantalite mineral group is the most common group of Nb and Ta bearing minerals. Tantalum is found together with Nb and Ti in at least 15 minerals, and with Nb in 12 minerals. Tantalum is the main impurity in commercial purity Nb. Its oxide, Ta_2_O_5_, has two polymorphs, a low temperature one (known as L-Ta_2_O_5_) and the high temperature H-Ta_2_O_5_. Their structure involves highly distorted TaO_7_ and TaO_6_ polyhedra [65] and the periodicity of the structure is sensitive to small concentrations of dopants [72]. Nb_2_O_5_ can form a solid solution with Ta_2_O_5_ and (Ta_1−x_Nb_x_)_2_O_5_ solid solutions crystallize at a lower temperature than Ta_2_O_5_.

To summarize, the atomic sizes of the elements that participate in the mixed oxides formed on Nb-silicide based alloys are important because the oxide structures consist of blocks of the ReO_3_ type with networks of octahedral MO_6_ groups.

Ta_2_O_5_ contains a large number of oxygen vacancies [73]. The loss of oxygen can be suppressed by TiO_2_ substitution [74]. The TiO_2_ addition significantly slows down the H- to L- phase reversion [72]. Ti substitution in the octahedra is more energy favorable [72]. By doping of TiO_2_ in Ta_2_O_5_, oxygen vacancies are compensated by Ti^4+^ ions that are substitutionally incorporated at Ta^5+^ sites [72]. The Ti^4+^ has radius similar to Ta^5+^, which allows for dense packing in the Ta and O matrix. 

The TiTa_2_O_7_ is isostructural with TiNb_2_O_7_ and Nb and Ta are compatible in rutile. The latter is often considered as stoichiometric TiO_2_ and Nb^5+^ and Ta^5+^ replace Ti^4+^ in the octahedral Ti sites. The charge balance in these sites and interstitial sites is provided by oxygen vacancies. The diffusivities of Ti and oxygen in rutile are significantly higher than those of Nb and Ta that are also higher than the diffusivity of Hf in rutile. Furthermore, the diffusivity of Nb in rutile is four times that of Ta [75]. Marschall et al. [75] attributed the high D_Nb_^TiO2^/D_Ta_^TiO2^ ratio to differences in electronegativity rather than to mass difference after considering the factors that influence the substitution of elements in minerals, namely ionic charge (the same for Nb and Ta), ionic radii (very similar for Nb and Ta) and electronegativity (1.6 for Nb vs. 1.5 for Ta). The latter influences bond strength and thus the potential for substitution in rutile [75].

The above discussion shows that in the oxidation of Nb-silicide based alloys there is inter-dependence of solubility and diffusivity of oxygen, oxidation and diffusion of Nb and solute elements and chemistry and structure of the oxides on atomic size, electronegativity and VEC.

### 2.2. Creep

In polycrystalline alloys, creep is controlled by diffusion-controlled dislocation creep, grain boundary sliding and diffusional creep. The creep rate έ is related to the stress σ with the power law relation έ ∝ σ^n^. Harper-Dorn creep occurs when n = 1. The exponent n can be approximately equal to 3 (viscous glide of dislocations, activation energy Q_creep_ approximately equal to the activation energy for interdiffusion of solute atoms Q_interdiffusion_), or 5 (climb of dislocations) or 8 (dislocation climb creep under constant microstructure). For the latter two cases Q_creep_ is approximately equal to the activation energy for self-diffusion Q_self-diffusion_. Creep data for dispersion hardened alloys provides support for n ≈ 8. However, the latter alloys can have Q_creep_ ≠ Q_self-diffusion_. Metal matrix composites can have high and variable stress exponent and activation energy, but when a threshold stress (stress below which creep cannot occur) is considered the values of the above parameters are similar to those of n and Q_creep_ of pure metals and solid solution alloys [76].

For Nb and the group 5 and 6 transition metals in the periodic table that can be in solution in Nb_ss_, often Q_creep_ is noticeably less than Q_self-diffusion_ for homologous temperatures less than 0.6 and often much higher at higher homologous temperatures, and the values of n can be in the range 4 to 7 [77]. For intermetallics that can be present in the microstructures of Nb-silicide based alloys the following is known regarding creep. The compressive creep of NbCr_2_ at 1200 °C and σ = 69 MPa gave n = 2 and Q_creep_ ≈ 130 kJ/mol [64]. The compressive creep of Nb_3_Al at 1000 °C was comparable with that of Ni based superalloys at the same temperature and gave n = 2.86 and Q = 350 kJ/mol [78]. The latter is close to the activation energy for chemical inter-diffusion in Nb_3_Al (Q ≈ 366 kJ/mol [79]) and the activation energy for the diffusion of Al in Nb (335 kJ/mol [6]). For the creep of Nb_3_Al at 1200 °C the stress exponent n was 2.19 [78]. For Nb_3_Al, nano-indentation creep gave creep exponent 4.2 and Q = 531 kJ/mol, or about 1.5Q_self-diffusion_ [79]. The compressive creep of Nb_3_Sn (for 25 at.% Sn) at 1400 °C gave n = 4.5 and Q ≈ 450 kJ/mol [78] (compared with 320 kJ/mol for the activation energy for the diffusion of Sn in Nb [6]). The creep at 850 and 1000 °C of dual phase alloys based on (Ti,Nb)_5_(Si,Al)_3_ and (Ti,Nb)_3_(Si,Al) could be described in terms of power law creep [80].

The Nb-silicide based alloys are also known as in situ composites where the two most important phases are considered to be the bcc Nb_ss_ and tetragonal Nb_5_Si_3_ (see introduction). Experimental data has given n ≈ 1 for the Nb_5_Si_3_ silicide and n ≈ 3 for the Nb_ss_ [16,17] (see previous paragraph for n values of other intermetallics that can be present in the microstructures of Nb-silicide based alloys). The volume fractions of these phases can differ between composites. When the matrix is the strong phase (silicide) the composite is an (ductile phase toughened) intermetallic matrix composite (IMC). When the matrix is the weak phase (Nb_ss_) the composite is a metal matrix composite (MMC). Either composite type can have more than one type of bcc Nb_ss_ (see [6]) and Nb_5_Si_3_ silicide (meaning βNb_5_Si_3_ and/or αNb_5_Si_3_ and/or γNb_5_Si_3_, see introduction), the silicide can have complex composition with Nb and Si substituted by other solute elements (see example given in the introduction and [47]) and there is partitioning of Ti and Hf at the interface between Nb_ss_ and silicide [9,47]. The latter interface is rich in Ti and Hf, during exposure to high temperature only the solid solution is homogenized but the interface between solid solution and Nb_5_Si_3_silicide can continue to be rich in Ti but only on the silicide side [9]. Furthermore, there can be precipitation of second phase(s) in the silicide [5,7,9,81,82]. Alloying affects the creep of all phases (see below), can stabilize the hexagonal γNb_5_Si_3_ silicide (which is considered undesirable for creep [1]), can result to significant changes of the properties of tetragonal Nb_5_Si_3_ [47] and can promote or slow down the transformation of βNb_5_Si_3_ to αNb_5_Si_3_ (for example, see [5,7,9,81,82]). A consensus has not been reached about the type(s) of other intermetallic phases that are desirable for the creep of Nb-silicide based alloys. Research on how different microstructures affect the creep of Nb-silicide based alloys is also extremely limited, owing (i) to the limited resources worldwide for the processing of these materials and (ii) the limited availability of material for experimental work (see introduction).

The parameters δ, Δχ and VEC, which are based respectively on atomic size, electronegativity and valence electron concentration and describe the alloying behavior of Nb-silicide based alloys and the phases that are present in their microstructures [6,41,46,47,48], depend on electronic structure. Could these parameters be related to creep rate? *Creep* refers to the plastic deformation of a material with time at constant stress. Creep theories consider the motion of dislocations in the microstructure. The creep rate έ is related to the dislocation velocity υ (έ = bρυ, υ = Bτ_eff_^m^exp(−U/*k*T), where ρ is the density of mobile dislocations, b is Burger’s vector, τ_eff_ is an effective shear stress acting on the dislocation, B is a constant and m and U describe the stress and temperature dependence of the dislocation velocity [83]). What is the role played by electronic structure in the motion of dislocations in plastic flow? Below, deformation is briefly considered in the context of dislocations and electronic structure.

The deformation of materials can be dislocation mobility controlled (intrinsic resistance to dislocation motion) or dislocation obstacle controlled (extrinsic resistance). Materials in which flow is limited by dislocation mobility are strain rate sensitive and are characterized by a low stress exponent n and a large strain rate exponent. In such materials dislocations propagate by the motion of kinks, in other words the latter limits dislocation mobility. In materials where flow is dislocation obstacle controlled, deformation occurs by the rapid motion of highly mobile dislocations that are free to glide until they become obstructed by extrinsic resistances such as the dislocation substructure, precipitates and phase interfaces and other obstacles (see below), at which point the generation of new dislocations is required to continue deformation. Extrinsic resistances are those in which dislocation lines as a whole interact with some structural feature of a material and include the cutting or bypassing of precipitates (Orowan bowing) by dislocations.

The deformation of bcc metals and Class I solid solutions is controlled by dislocation mobility, i.e., by the motion of kinks. In contrast, in pure fcc metals and Class II solid solution alloys dislocation mobility is controlled by obstacles such as dislocation substructure. The activation volume (V_activation_) and the stress exponent n can be used to identify the microstructure mechanisms that control deformation. For the bcc metals Nb and W the stress exponent n respectively is in the range 5 to 7 and 5 to 9 and the activation volume V_activation_ respectively is 50**b^3^** and 5**b^3^** (b is Burger’s vector) [84] compared with the significantly higher n and V_activation_ values for metals whose flow is dislocation obstacle controlled. For example, for Cu, n = 100 and V_activation_ = 2000**b^3^** [85]. For Si and Ge (covalent bonding) the V_activation_ values at 900 °C were 23.4**b^3^** and 18**b^3^** respectively and 9**b^3^** for Ge at 600 °C [86].

The effect of symmetry on bonding is very important. The band gap between the conduction and valence energy bands forms a basis for properties such as elastic stiffness, plastic flow resistance (dislocation mobility), and crystal structure stability. The larger the band gap the more stable the crystal structure. Dislocation mobilities determine rates of plastic shape change. The arrays of atoms at the cores of dislocations are important for mechanical behavior. The symmetry changes as material passes into and then out of the core of a moving dislocation. Dislocation lines move through kinks that lie along their lengths. The atomic configuration at a kink is very different than it is in the normal crystal structure. Kink mobility is directly related to electronic structure. The activation energy for dislocation mobility in Si, Ge and Sn is proportional to the band gap. In Si (covalently bound element) kinks are very localized [87]. The mobility of dislocations is very small in covalently bonded crystals like Si.

The range of dislocation mobilities is very large when measured by the stress needed to move a dislocation. This range is from very small in a perfect metal to about G/4π for a covalently bonded crystal (G is the appropriate shear modulus). Extrinsic resistance to dislocation mobility arises from dislocations, stacking faults, vacancies, interstitials, grain boundaries, anti-phase boundaries, twins, precipitates, free surfaces and others. Theories of creep in metals and alloys consider extrinsic resistances and grain size. Dislocations move by becoming intermittently trapped by various obstacles followed by “free run”. The former is caused by extrinsic factors (see above) that create obstacles to dislocation movement. The latter is limited by intrinsic factors, i.e., by the interaction of dislocations with conduction electrons and phonons.

Dislocation mobility depends strongly on chemical bonding. In simple metals the theoretical width of a dislocation is a few atomic spaces but the bonding is very delocalized, thus the energy of a dislocation is nearly independent of its position. In covalently bonded crystals the bonding is highly localized to the regions between pairs of atoms (less than one atomic distance) and this depends strongly on the position of the center of a dislocation. In the transition metals the electrons that contribute most to the cohesion are localized in spd-hybrid bonds. Thus, the cohesive energy is not nearly independent of the atomic configuration.

In bcc metals slip occurs in the <111> direction and the Burger’s vector is α/2<111>. The bonding in bcc metals gives the screw dislocations a non-planar core structure. Dislocations move through the nucleation and propagation of double kinks. There is disagreement about the fundamental slip planes in bcc metals, i.e., the planes where kinks form. According to slip trace analyses at low temperatures slip always occurs on {110} planes and as the temperature increases slip is observed on {110}, {112} and {123} planes in order of increasing rarity. The continuum theory predicts slip on {110} planes at low temperatures and on {112} for temperatures above about 100 K but atomistic simulations show {110} slip at 0 K and at finite temperatures [88].

For edge and screw dislocations the bonding disregistry respectively is perpendicular to and along the dislocation line direction. The elastic energy of a dislocation is independent of the sense of its Burger’s vector but its mobility need not be because its core may not have mirror symmetry about its mid-point. In bcc metals the mechanical properties are governed by ½<111> screw dislocations, and asymmetry appears when the glide direction is <111>. The nature of the dislocation core dictates the types of kinks that can form on a dislocation line. Dislocation kink mechanisms are linked with symmetry breaking that can be caused by mixed character of dislocation, the symmetry of the crystal lattice and atomic core reconstruction. The latter is dependent on the details of interatomic interaction [89]. Atomistic simulations of screw dislocation cores in Mo and Ta have shown them to be non-degenerate cores. There is no data for the screw dislocation cores in Cr, Nb and V but it is expected that they are also non-degenerate [88]. In other words, dislocation cores in bcc metals are non-degenerate and spatially spread. The spreading into {110} planes varies locally depending on local atomic composition [90]. An atomistic modelling study of kinks on screw dislocation in Si concluded that “the structure of a single kink is characterized by a narrow core and highly stretched bonds between some of the atoms”. Ge has similarities with Si regarding dislocation properties [91].

Correlations exist between the cohesive properties and electronic structure band. Covalently bonded solids possess intrinsic plastic resistance. The motion of dislocations is limited by the motion of their cores and the core motion is limited by the motion of kinks along the cores. In materials with localized bonding, dislocations are expected to move bond by bond. At a kink the chemical structure is severely disrupted (a chemical bond is broken). This disruption is very localized. 

In the microstructures of Nb-silicide based alloys the bcc Nb solid solution(s) co-exist with covalently bound compounds (silicides and other intermetallic compounds, see introduction). The importance of electronic structure for the properties of intermetallic compounds in Nb-silicide based alloys was demonstrated in [47,48] using the parameters VEC and Δχ. The latter is related to electronegativity and the former, which gives the number of valence electrons per atom filled into the valence band, is key to determining the Fermi level in the valence band [56]. Changes in the properties of the intermetallic phases were related to the parameters VEC and Δχ [47,48]. For example, the worsening of the creep of alloyed Nb_5_Si_3_ compared with the unalloyed Nb_5_Si_3_ was accompanied by decrease of VEC and increase or decrease of Δχ depending on alloying addition(s) [47], and the better creep of Nb(Cr,Si)_2_ Laves phase compared with the unalloyed NbCr_2_ Laves phase was related to the decrease of the VEC and Δχ parameters [48]. In [41] it was shown that the hardness of microstructures containing both Nb_ss_ and Nb_5_Si_3_ or Nb_ss_, Nb_5_Si_3_ and A15-Nb_3_X increased as the VEC parameter of such microstructures increased. The same trend between hardness and VEC was observed for the hardness of the A15-Nb_3_X phases in the Nb-silicide based alloys [48], and for β(Nb,Ti)_5_Si_3_ and alloyed tetragonal Nb_5_Si_3_ [41]. The strong relationship between the hardness and VEC of eutectics with Nb_ss_ and Nb_5_Si_3_ was attributed to the covalent bonded intermetallic phase(s) in the eutectics, with the latter being the key phases that determined the hardness of the eutectics [41]. Most importantly, the trends between the C_44_ and VEC and hardness and VEC of α(Nb,Ti)_5_Si_3_ were the same as those reported for transition metal covalently bonded carbonitrides [41]. Also the trend between the C_44_ and VEC of β(Nb,Ti)_5_Si_3_ was the same as that reported for covalently bonded M_2_AlC compounds [41].

Dislocation mobility is fundamental to mechanical behavior and plastic deformation of materials including creep. How atomic size, electronegativity and valence electron concentration can elucidate the creep of Nb-silicide based alloys? What role can the electronic structure play in the creep of Nb-silicide based alloys? Are mechanisms related to electronic structure (i.e., intrinsic mechanisms) important in the creep of Nb-silicide based alloys? The following discussion will make a case that the link between the creep of Nb-silicide based alloys and their parameters δ (related to atomic size), Δχ and VEC, which relate to the electronic structure of alloys, is attributed primarily to the covalently bound intermetallics in their microstructures and to the increase of the covalency of the Nb_ss_ with alloying. In Section 2.3 it will be shown that there exist relationships between creep rate and each of the above parameters. 

The electronic structure that underlies the structural geometry of elements plays a key role in determining the mechanical behavior of metals and alloys. Chemical bonding, which is provided by electronic structure, is crucial to mechanical behavior. The sizes of atoms are determined by energies of electrons in occupied quantum states. Interactions between atoms provide cohesion via the redistribution of the bonding electrons between the atoms. The spatial distribution of bonding electrons is key to mechanical behavior. 

The elastic constants depend on structural geometry and the corresponding electronic structure. The shear coefficients are the most important sub-sets of the elastic constants. The Young’s modulus is a function of the bulk modulus B and the shear modulus G. The latter rather than the former appears in the equations of dislocation theory. Both B and G are fundamental coefficients for describing mechanical behavior.

The primary factor that determines elastic stiffness is chemical constitution because the latter determines bonding. The covalent bonds are the stiffest. A key parameter in bonding is the size of atoms (bond length). A key parameter for elastic stiffness is the valence electron concentration. In covalent bonds the charge associated with electron pairs is localized. In metallic bonds the bonding electrons are delocalized. 

The shear moduli depend on both the shear plane and the shear direction and the structures of both of these depend on crystal symmetries and local atomic structure. For simple metals (those bonded primarily by s- and p-level electrons) as the number of valence electrons increases the bulk stiffness increases, and decreases as the atomic size increases. Only the s and p quantum states contribute to the cohesion of these elements. For the transition metals the occupied d and f states play an important role in the cohesion. The valence electron concentration is important for the bulk modulus of the covalently bonded Ge, Si and Sn. In covalently bonded materials the shear moduli can be significantly larger compared with the bulk moduli.

**Figure 1:**
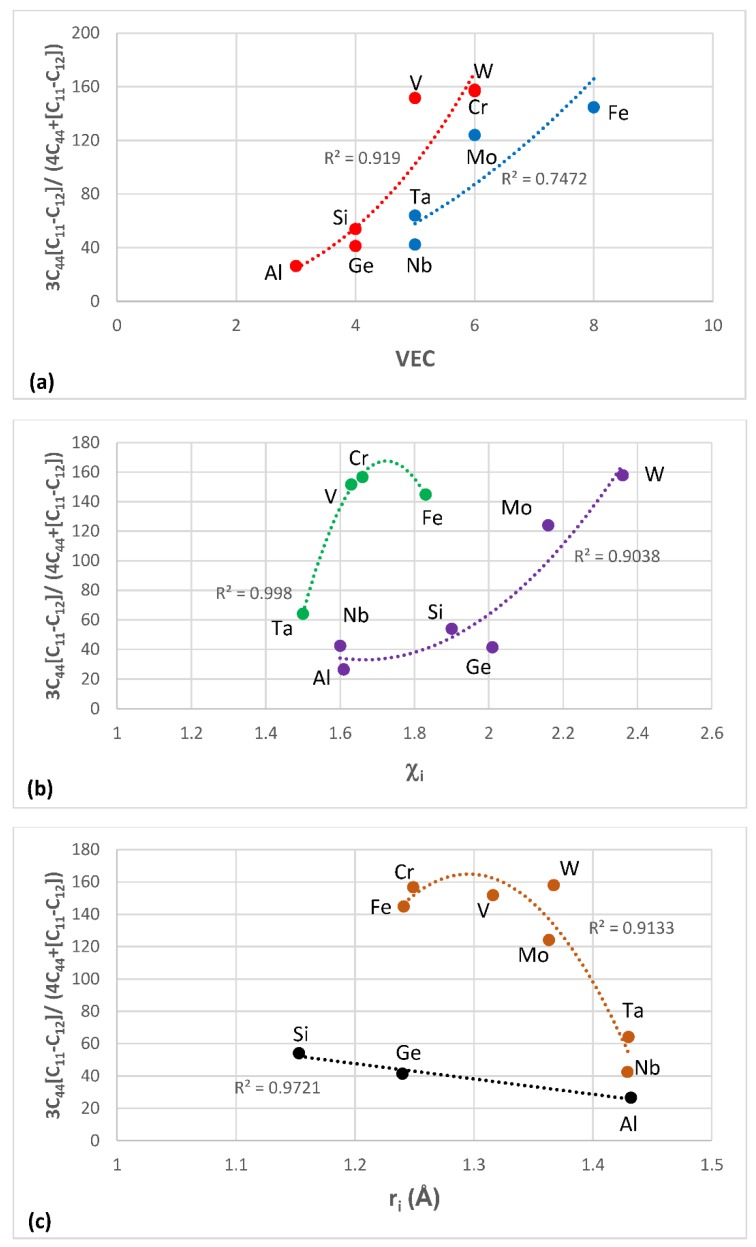
Plots of 3C_44_[C_11_-C_12_]/(4C_44_ + [C_11_-C_12_]) versus (**a**) VEC; (**b**) χ_I_ and (**c**) r_i_ for cubic metals in Nb-silicide based alloys.

**Figure 2:**
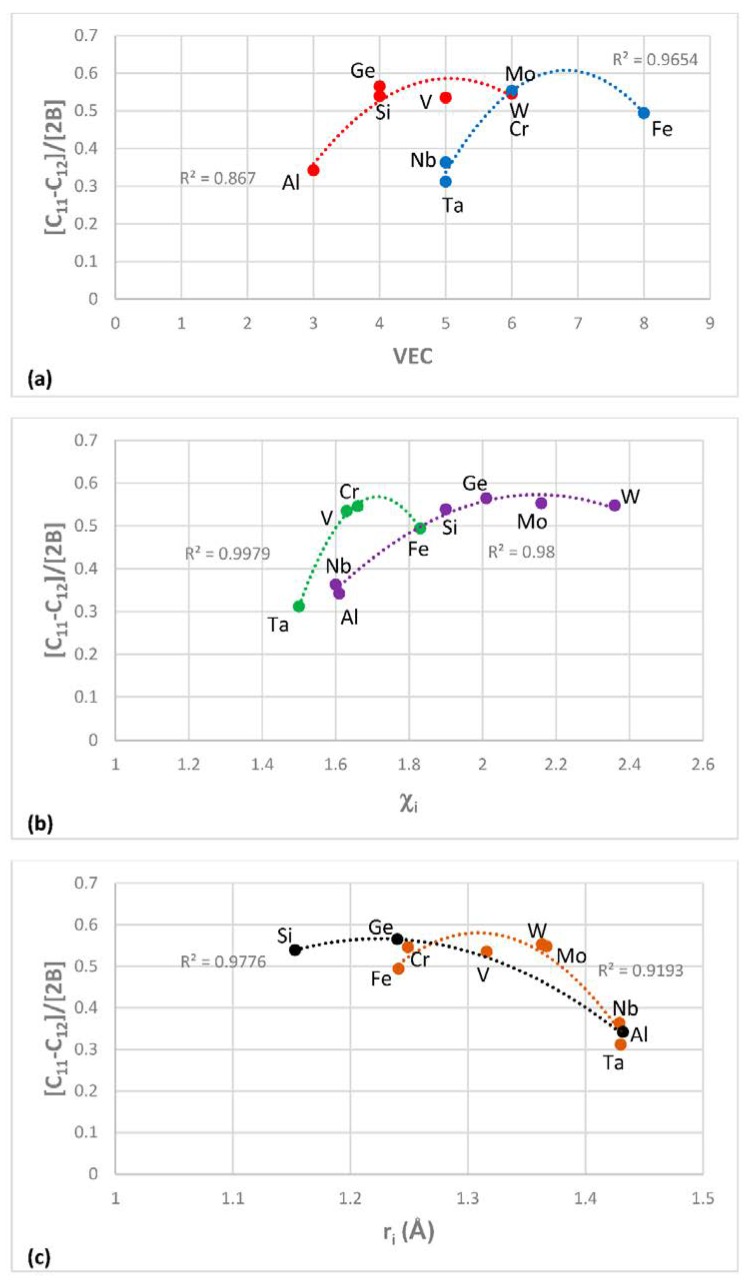
Plots of [C_11_-C_12_]/[2B] versus (**a**) VEC; (**b**) χ_I_ and (**c**) r_i_ for cubic metals in Nb-silicide based alloys.

**Figure 3:**
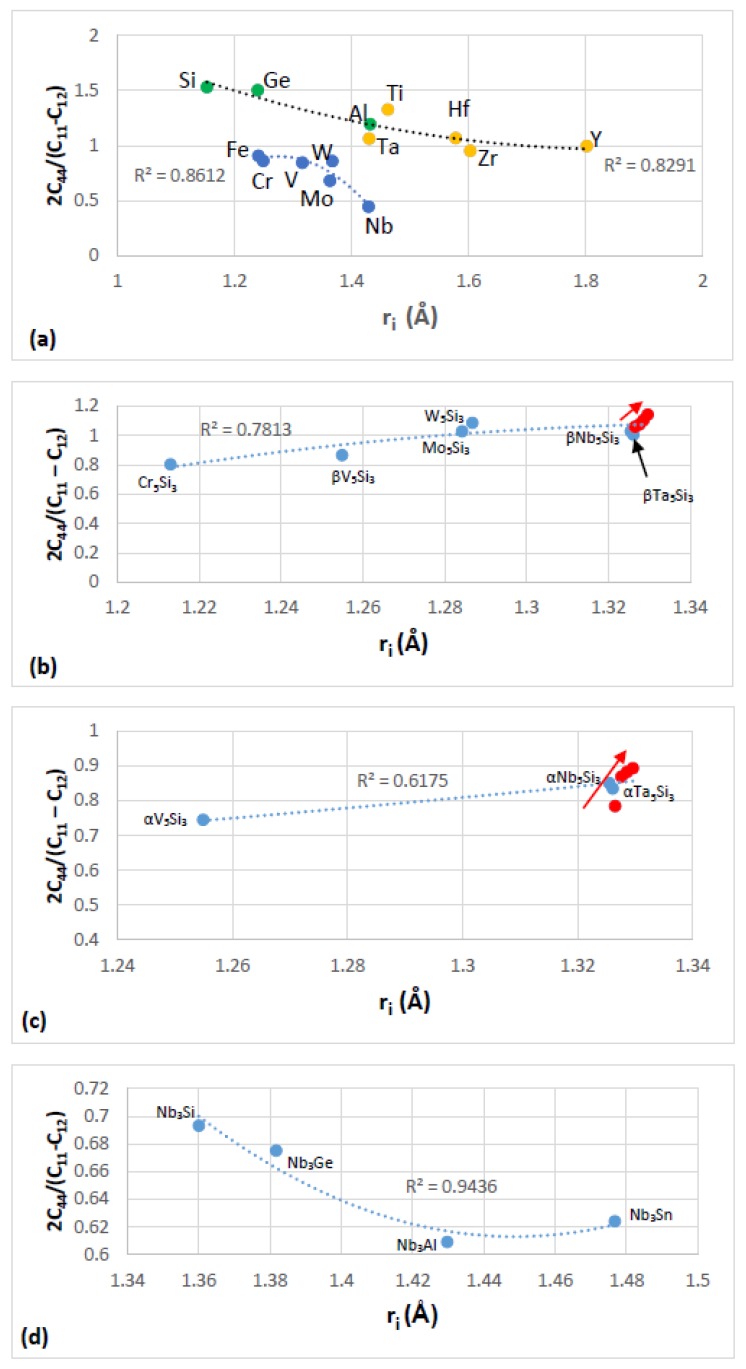
Plots of 2C_44_/(C_11_-C_12_) for (**a**) cubic and hexagonal metals; (**b**) 5-3 silicides with W_5_Si_3_ prototype; (**c**) 5-3 silicides with Cr_5_B_3_ prototype and (**d**) A15 compounds. Red color data points are for alloyed (Nb,Ti)_5_Si_3_, see text. In (**b**,**c**) the red arrows point from low to high Ti concentration in (Nb,Ti)_5_Si_3_, i.e., from 3.125 to 12.5 at.% Ti.

**Figure 4:**
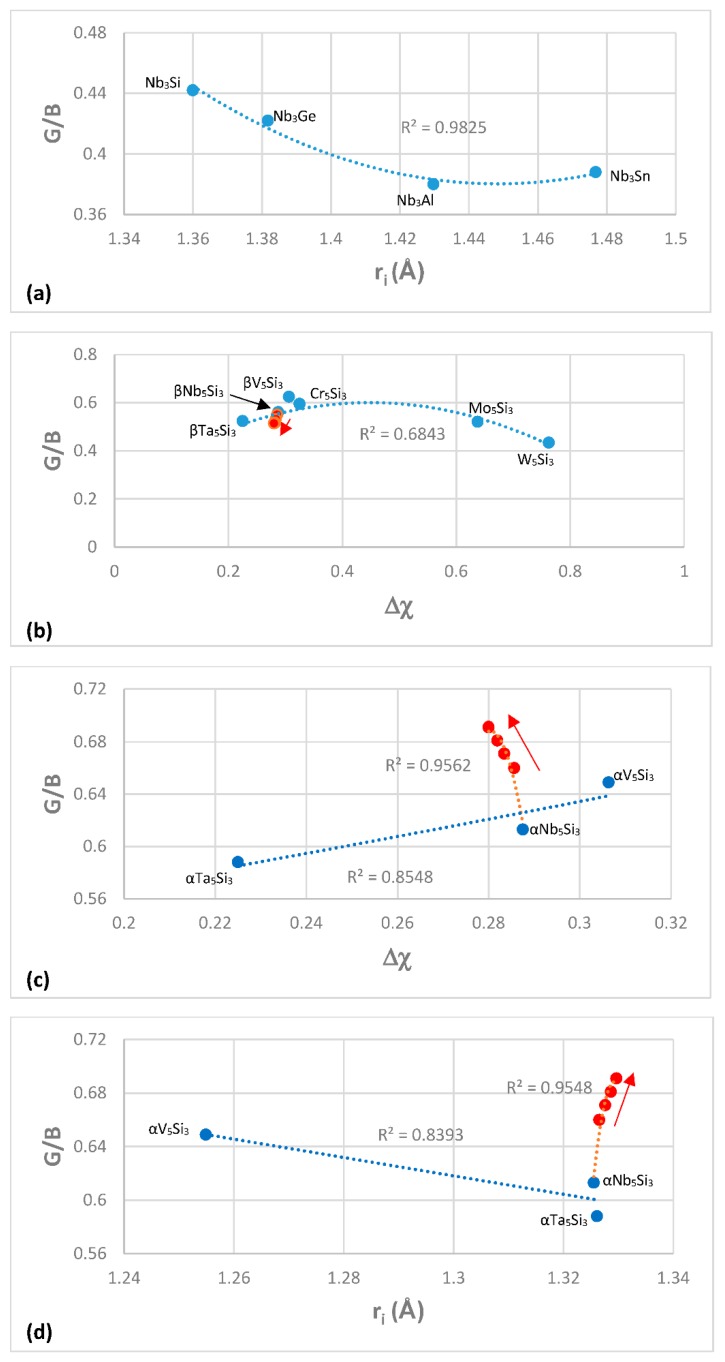
Plots of G/B versus r_i_ for (**a**) A15-Nb_3_X compounds and (**d**) 5-3 silicides with Cr_5_B_3_ prototype and G/B versus Δχ for (**b**) 5-3 silicides with W_5_Si_3_ prototype and (**c**) 5-3 silicides with Cr_5_B_3_ prototype. Red color data points are for (Nb,Ti)_5_Si_3_, see text. In (**b**–**d**) the red arrows point from low to high Ti concentration in (Nb,Ti)_5_Si_3_, i.e., from 3.125 to 12.5 at.% Ti.

The most characteristic shear moduli for cubic symmetry are the C_44_ (shear on (100) planes), (C_11_-C_12_)/2 (shear on (110) planes in the face diagonal directions) and 3C_44_(C_11_-C_12_)/[4C_44_ + (C_11_-C_12_)] (shear on (111) planes). Figure 1, Figure 2, Figure 3 and Figure 4 show data for shear moduli, Zener anisotropy factor (see below) and G/B ratios of Nb and other cubic and hexagonal metals in Nb-silicide based alloys, 5-3 silicides and A15 compounds. The lines in these figures are provided to highlight trends for different groups of metals and compounds.

The shear moduli of metals of cubic symmetry that are alloying additions in Nb-silicide based alloys belong in different groups when the aforementioned characteristic shear moduli are plotted versus VEC, Pauling electronegativity χ_i_ and atomic size r_i_. Figure 1 shows plots of the latter of the above moduli parameters versus VEC, χ_i_ and r_i_. The same groups of elements as in Figure 1a–c respectively were in plots of C_44_ versus VEC and (C_11_-C_12_)/2 versus VEC (not shown), in plots of C_44_ versus χ_i_ and (C_11_-C_12_)/2 versus χ_i_ (not shown) and in plots of C_44_ versus r_i_ and (C_11_-C_12_)/2 versus r_i_ (not shown).

The G/B ratio is positive, is small for ductile materials and increases as the materials becomes increasingly rigid and more brittle. For covalently bonded solids the ratios C_44_/B, [C_11_-C_12_]/[2B] and 3C_44_(C_11_-C_12_)/[B(4C_44_ + (C_11_-C_12_))] are high and are greater than one for diamond (about 1.3, 1.08 and 1.14 respectively [92]). In such solids the dislocation mobility is significantly reduced compared with elements with metallic bonding. Increased covalency due to alloying would result to decreased dislocation mobility. Figure 2 shows plots of the second of the above ratios versus VEC, χ_i_ and r_i_. The same groups of elements as in Figure 2a–c respectively were in plots of C_44_/B versus VEC and 3C_44_(C_11_-C_12_)/[B(4C_44_ + (C_11_-C_12_))] versus VEC (not shown), in plots of C_44_/B versus χ_i_ and 3C_44_(C_11_-C_12_)/[B(4C_44_ + (C_11_-C_12_))] versus χ_i_ (not shown) and in plots of C_44_/B versus r_i_ and 3C_44_(C_11_-C_12_)/[B(4C_44_ + (C_11_-C_12_))] versus r_i_ (not shown). Note that the colours indicate the same groups of elements in Figure 1a and Figure 2a, Figure 1b and Figure 2b and Figure 1c and Figure 2c.

The Zener anisotropy constant A = 2C_44_/[C_11_-C_12_] gives the deviation from isotropy for cubic, tetragonal and hexagonal structures [93]. Figure 3a is a plot of this parameter versus atomic radius r_i_ of cubic and hexagonal symmetry elements in Nb-silicide based alloys. Note that the hexagonal metals Hf, Ti, Y and Zr are in the same group with Al, Ge, Si and Ta. Also note that the grouping of elements in Figure 1, Figure 2 and Figure 3a is in accordance with the separation of Nb-silicide based alloys in different groups that was discussed in [46]. The grouping of elements would also indicate that if there were to be relationships between mechanical properties and alloy parameters VEC, δ and Δχ, they would apply for specific groups of elements in Nb-silicide based alloys. The data for C_11_, C_12_ and C_44_ for the elements in Figure 1, Figure 2 and Figure 3a is from [94] (Al), [95] (Cr), [96] (Fe, V), [97] (Ge), [98] (Mo), [99] (Nb, Si), [100] (Ta), [101] (W) and [102] (Hf, Ti, Y, Zr).

Data for the Zener parameter A for 5-3 silicides with prototypes W_5_Si_3_ or Cr_5_B_3_ is plotted versus atomic radius respectively in Figure 3b,c and for A15-Nb_3_X compounds in Figure 3d. The R^2^ values in Figure 3b,c correspond to all the data. Note that the plots based on atomic radius show that the effect of alloying on the Zener parameter of tetragonal Nb_5_Si_3_ is weak. The metalloid elements form covalent bonds with metals in intermetallic compounds, which tend to reduce the dislocation mobility in these compounds. The latter have negligible ductilities because of the low mobilities of dislocations. Figure 4 shows plots of G/B for A15-Nb_3_X compounds and 5-3 silicides with prototypes W_5_Si_3_ or Cr_5_B_3_. The G/B data is plotted versus atomic radius in Figure 4a,d and versus Δχ in Figure 4b,c. Note similar trends in Figure 3d and Figure 4a for the A15 compounds. Also note that the plots of G/B versus r_i_ or Δχ show clearly the strong effect of alloying with Ti on increasing the G/B ratio and thus the covalency of α(Nb,Ti)_5_Si_3_. Figure 4c,d also show that alloying with Ti has a stronger effect on the covalency of αNb_5_Si_3_ compared with βNb_5_Si_3_. The data for G/B and C_11_, C_12_ and C_44_ for the 5-3 silicides and A15-Nb_3_X compounds is from [103] (Cr_5_Si_3_), [99] (Nb_5_Si_3_), [104] ((Nb,Ti)_5_Si_3_), [100] (Mo_5_Si_3_, Ta_5_Si_3_), [105] (W_5_Si_3_, V_5_Si_3_), [106] (Nb_3_Sn) and [107] (Nb_3_Si).

Elastic properties like Young’s modulus and shear modulus depend on actual composition. Chan reported that the Young’s modulus of tetragonal unalloyed Nb_5_Si_3_ is reduced when Nb is substituted by Ti and as the concentration of Ti in (Nb,Ti)_5_Si_3_ increases (the structure changes to hexagonal (Ti,Nb)_5_Si_3_) the modulus decreases further towards that of Ti_5_Si_3_ [108]. Papadimitriou et al. [104] showed that this is the case only of βNb_5_Si_3_ and that the substitution of Nb by Ti in αNb_5_Si_3_ increases the Young’s modulus.

The shear moduli increase relatively more than the bulk moduli; thus, the G/B ratio is greater for intermetallic compounds compared with metals. For example, for Nb the G/B ratio is 0.228, for βNb_5_Si_3_ this ratio is 0.54 and for αNb_5_Si_3_ is 0.613. In other words, the tetragonal Nb_5_Si_3_ is more than twice as rigid as Nb. For the Nb solid solutions with no Sn or B additions that were given in the Table 1 in reference [6] the G/B ratio increases with alloying and is 54% higher than that of pure Nb for the 40.4Nb-31.5Ti-1.6Si-2.7Hf-1Ge-15Cr-7.8Al solid solution, which is about 90% of the G/B ratio of γNb5Si3 [104]. Alloying can increase further the G/B ratio of the Nb_ss_, for example in one alloy recently studied in our group [109] the G/B ratio of the Nb_ss_ with no Si was 0.403, i.e., higher than that of γNb_5_Si_3_ (the G/B ratios of the aforementioned Nb_ss_ were calculated using the rule of mixtures). These high G/B ratios would also suggest that the Nb_ss_ becomes increasingly rigid and more brittle as the ratio is increased. The larger G/B ratios of the tetragonal Nb_5_Si_3_ and alloyed solid solution indicate that the dislocation core energies depend strongly on their positions.

The location of the Fermi level is indicative of phase stability. For intermetallics, a pseudo-gap in the density of states is observed close to the Fermi level owing to the combined effects of charge transfer and hybridization. For example, when there is large electronegativity difference between elements, the redistribution of electrons changes the shape of the band, the screening electrons are assigned to low states in the band and this gives the minimum in the density of states curve. The intermetallic is stable when the Fermi level is exactly at the pseudo-gap and unstable in the antibonding region (Fermi level to the right of the pseudo-gap). Alloying may stabilize a metastable intermetallic phase. When the Fermi level is to the left of the pseudo-gap (bonding states) not all bonding states are completely filled and additional electrons are needed to increase stability. If the Fermi level falls on a peak in the density of state curve of the intermetallic the D_self-diffusion_ of the latter is increased. Considering the Ti and Hf rich Nb_ss_/Nb_5_Si_3_ interfaces in Nb silicide-based alloys and their contamination by oxygen and formation of hafnia near Hf rich areas of the Nb_5_Si_3_ [15,57,81], the changes of the Fermi level resulting from alloying will affect the stability of phases and their properties, both of which are important in creep.

**Figure 5:**
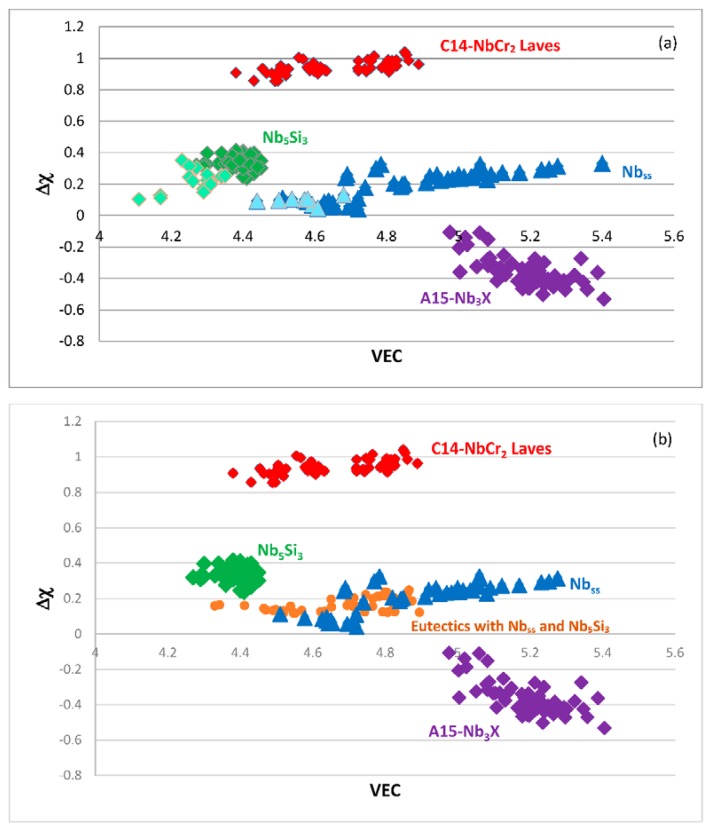
Maps of Δχ (ordinate) versus (abscissa) valence electron concentration (VEC) (**a**) of Nb_ss_ (blue triangles), Nb_5_Si_3_ (green diamonds), C14-NbCr_2_ Laves (red diamonds) and A15-Nb_3_X (purple diamonds) phases where Boron containing Nb_ss_ and Nb_5_Si_3_ are shown in light blue and light green and (**b**) of Nb_ss_ (blue triangles), Nb_5_Si_3_ (green diamonds), eutectics with Nb_ss_ and Nb_5_Si_3_ (orange circles), C14-NbCr_2_ Laves (red diamonds) and A15-Nb_3_X (purple diamonds) phases.

**Figure 6:**
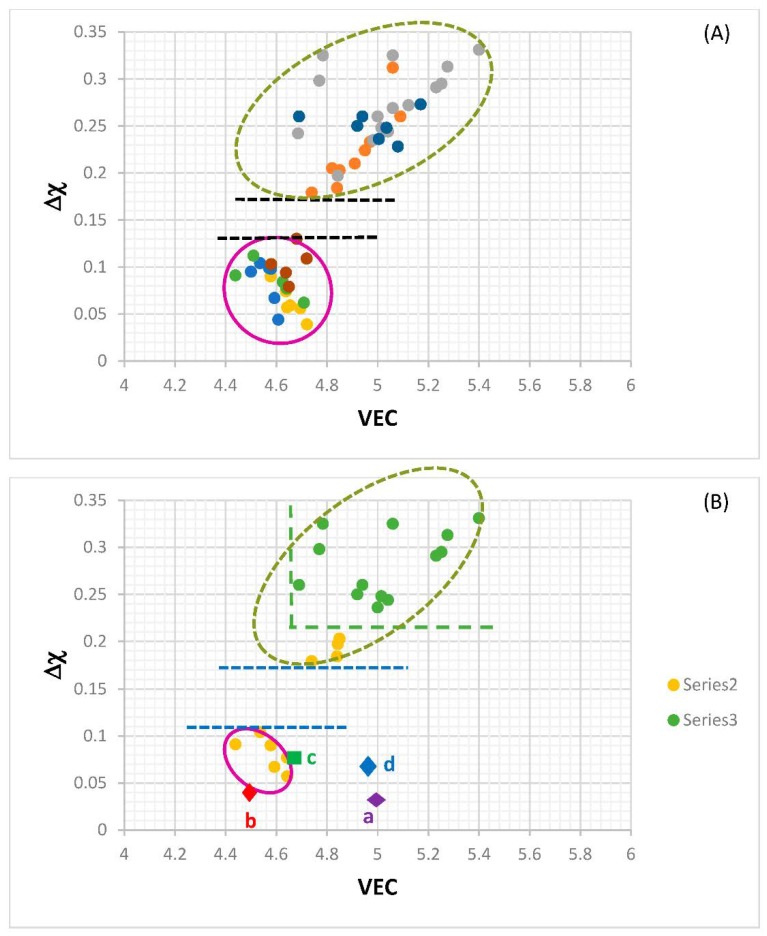
Maps of Δχ (ordinate) versus (abscissa) VEC for the Nb_ss_, (**A**) data for all solid solutions and (**B**) data for Nb_ss_ with no Si and Nb_ss_ rich in Ti (see [6]). In (**B**) the series 2 data are for the Nb_ss_ rich in Ti and the series 3 is for the Nb_ss_ with no Si, see text. For the gap in Δχ values see [6]. See Figure 7C and text for the alloys represented by a, b, c and d in (**B**).

In reference [47] it was shown that the alloying of Nb_5_Si_3_ changed the position of the Nb_5_Si_3_ silicide in Δχ versus VEC maps and that the changes (meaning increase or decrease) of these parameters depended on specific alloying addition(s). The effect of the substitution of Nb by Ti was clearly demonstrated, as was the substitution of Si by Ge or Sn and the alloying of the silicide with B in the Figures 5 and 6 in reference [47]. The specific case of the substitution of Nb only with Ti in Nb_5_Si_3_ silicides was studied using first-principles calculations in [104] to find out how the stability of different Nb_5_Si_3_ silicides and their elastic properties are affected with increasing Ti concentration. It was shown that for all 5-3 silicide structures (meaning tetragonal αNb_5_Si_3_ and βNb_5_Si_3_ and hexagonal γNb_5_Si_3_) the main contribution to the total electronic density of states (TDOS) was the partial electronic density of states (PDOS) of d electron states, followed by the p electron states. The s electron states contributed the least to the TDOS of all structures. For the unalloyed Nb_5_Si_3_ silicide the gradual decrease of phase stability from tetragonal to hexagonal Nb_5_Si_3_ silicide was explained by the location of the Fermi level of each silicide. The hexagonal γNb_5_Si_3_ silicide became stable compared with the tetragonal αNb_5_Si_3_ and βNb_5_Si_3_ when the Ti concentration reached 50 at.%. For the αNb_5_Si_3_ and γNb_5_Si_3_ silicides the shear and Young’s moduli increased with increasing Ti addition and decreased in the case of βNb_5_Si_3_. The substitution of Nb by Ti strengthened atomic bonding in αNb_5_Si_3_ and γNb_5_Si_3_, and decreased bond strength in βNb_5_Si_3_. The above discussion shows that the Nb_ss_/Nb_5_Si_3_ interface in Nb-silicide based alloys is dynamic, meaning as the local chemistry changes during exposure to high temperature so do the mechanical properties of the interface.

The creep properties of Nb-silicide based alloys are key for their application at high homologous temperatures where diffusion is important. Diffusivities in the solid solution and silicide will depend on composition and in the case of the silicide will also depend on crystal structure. The latter can change as solutes partition to the silicide. The case for Ti was demonstrated in [104]. These changes also will be important in oxidation. Activation energies for creep may be strongly dependent on concentration(s) of impurities, for example contamination of Nb_ss_ (mainly) and silicide(s) by oxygen near the surface and below it and even in the bulk alloy microstructure is possible in Nb-silicide based alloys, depending on alloy composition [14,15,57]. Relationships between atomic radius and electronegativity of solute elements in Nb and their activation energies for diffusion and diffusivity at 1200 °C were discussed in [6].

The above discussion has indicated that if there were to be relationships between the creep of Nb-silicide based alloys and their parameters δ, Δχ and VEC, such relationships (i) would be about the contributions to creep rates made by intrinsic resistances not extrinsic ones and (ii) would not give the contributions made separately by intrinsic resistances to each parameter. In other words, any relationships between creep rate and parameters would be averaging the contributions of intrinsic resistances to creep that are expressed by a relationship between creep rate and a parameter. In the following parts of this paper it will be shown that as a matter of fact the available experimental data does give relationships between creep rates and each of the alloy parameters δ, Δχ and VEC.

#### Phases in Nb-Silicide Based Alloys: Δχ versus VEC Maps and Creep

The alloying behavior of bcc Nb solid solutions, tetragonal Nb_5_Si_3_, eutectics with Nb_ss_ and Nb_5_Si_3_, hexagonal C14-NbCr_2_ Laves phases and cubic A15-Nb_3_X compounds that are formed in Nb-silicide based alloys was studied, respectively in [6,41,47,48] and the data is summarized in the Δχ versus VEC maps in Figure 5. Figure 5a shows the phases without the eutectic. The data for the eutectic is included in Figure 5b, where the data for Nb_ss_ and Nb_5_Si_3_ alloyed with B has been excluded (eutectics with solid solution and 5-3 silicide are formed in B containing Nb-silicide based alloys, but currently there is no data about the actual chemical composition of these eutectics). Note that some data in Figure 5b for the eutectic is in the areas that were occupied by the data for B containing Nb_ss_ and Nb_5_Si_3_ in Figure 5a. Details of the solid solution area in the map are shown in the Δχ versus VEC map in the Figure 6. Figure 6A has the data for all solid solutions in cast and heat-treated alloys [6]. Figure 6B shows the data for the Ti rich Nb_ss_ and the Nb_ss_ with no Si [6]. The former is not stable after heat treatment.

There is a gap in Δχ values of the Nb_ss_ in Figure 6, which cannot be easily recognized in Figure 5, and no solid solutions fall in the range 0.13 < Δχ < 0.18. This gap in the Δχ values of the Nb_ss_ was discussed in [6]. The solid solutions with no B, Ta and V have Δχ > 0.18 and the solid solutions with no W have Δχ < 0.13. In Figure 6B in the bottom ellipse are the Ti rich Nb_ss_ with no refractory metals (RMs) (series 2). These solid solutions have 30 < Ti < 47 at.%, 9 < Cr < 16 at.% and 2 < Ti/Cr < 4. The Ti rich Nb_ss_ with RMs are in the bottom of the top ellipse. These solid solutions are lean in Mo and W and have 5 < (Mo + W) < 10 at.%, 3 < (Mo/W) < 5 and 2 < {Ti/(Mo + W)} < 6. In Figure 6B the Nb_ss_ with no Si (series 3) has 0.23 < Δχ < 0.33. This type of solid solution has 1 < (Mo/W) < 3, 0 < {Ti/(Mo+W)} < 1 and (Mo + W) > 14 at.%.

**Figure 7:**
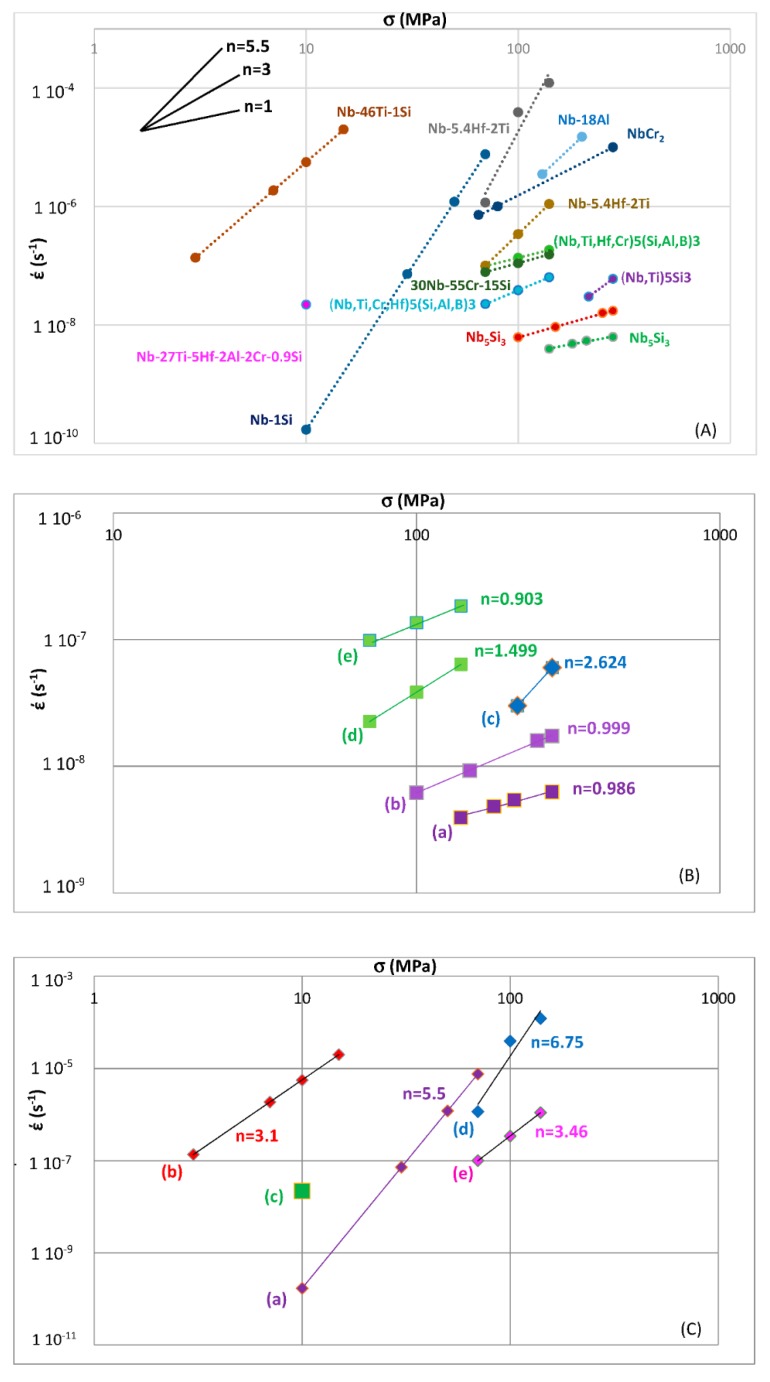
Norton plots for 1200 °C creep rate (s^−1^) (ordinate) versus (abscissa) stress (MPa), (**A**) for all types of phases in Nb silicide-based alloys namely unalloyed and alloyed Nb_5_Si_3_, NbCr_2_ Laves, Nb solid solution and Nb_3_Al (for 18 at.% Al); (**B**) for the 5-3 silicide and (**C**) for the bcc Nb solid solution. In (**B**) the data is for tetragonal 5-3 silicide where (a) αNb_5_Si_3_ [110], (b) αNb_5_Si_3_ [111], (c) (Nb,Ti)_5_Si_3_, (d) (Nb,Ti,Cr,Hf)_5_(Si,Al,B)_3_ and (e) (Nb,Ti,Cr,Hf)_5_(Si,Al,B)_3_ [111]. In (**C**) data for (a) Nb-1Si [108], (b) Nb-46Ti-1Si [112] (c) Nb-27Ti-5Hf-2Al-2Cr-0.9Si [112] (d) Nb-5.4Hf-2Ti for Q = 316 kJ/mol, see text and (e) Nb-5.4Hf-2Ti for Q = 374 kJ/mol, see text. The alloys in (**C**) are shown in Figure 2b.

Data for the creep of bcc Nb solid solutions, tetragonal Nb_5_Si_3_, hexagonal C14-NbCr_2_ Laves phases and cubic A15-Nb_3_Al are shown in Figure 7A and details of the Nb_5_Si_3_ silicide data and the Nb_ss_ data are shown respectively in Figure 7B,C. Note that the phases in Figure 5a are represented in Figure 7A. The creep data for the intermetallics was discussed in [47,48]. Figure 7A shows the gradual decrease of the value of the exponent n and the shift towards lower creep rates and higher stresses from the left-hand side (occupied by the solid solution) to the right hand side of the figure (occupied by the Nb_5_Si_3_ silicide). The values of the creep exponent n of the silicide and solid solution are given in Figure 7B,C, respectively. The n values for Nb_3_Al, NbCr_2_ and Nb-55Cr-15Si, respectively are 3.39, 1.8 and 0.99.

The Nb solid solutions for which there is creep data for 1200 °C (Figure 7C) are shown in Figure 6B. In Figure 7A,C the creep data for the Nb-1Si and Nb-46Ti-1Si solid solutions shows the adverse effect that Ti has on the creep of the solid solution. This is further supported by the data for the leaner in Ti solid solution of composition Nb-27Ti-5Hf-2Al-2Cr-0.9Si. The positions of the Ti rich solid solutions Nb-46Ti-1Si and Nb-27Ti-5Hf-2Al-2Cr-0.9Si with regard to the Ti rich Nb_ss_ are shown in Figure 6B. Notice that the solid solutions Nb-1Si and Nb-5.4Hf-2Ti are outside the area for the Ti rich Nb_ss_.

In Figure 7C there are two sets of data for the solid solution alloy Nb-5.4Hf-2Ti [113], which, as indicated in the caption, correspond to two different activation energies in έ = A(σ/E)^n^exp(−Q/RT), where E is the Young’s modulus [114]. The higher activation energy is closer to the activation energy for the diffusion of Hf in Nb [6]. The solid solution alloy Nb-5.4Hf-2Ti (known commercially as alloy C103) is considered as a Class I solid solution regarding its creep (n = 3). In this alloy, the Hf atoms rather than the Ti atoms were considered to be responsible for the solute effects on dislocation motion [113]. It has been suggested [113] that there is a critical concentration of Hf beyond which this element does not contribute significantly to strengthening at high temperatures. If the data for (d) and (e) in Figure 7C is correct, it would indicate a positive effect of Hf for the creep of Nb at low concentrations. 

Under creep conditions the constituent phases in Nb-silicide based alloys, namely the Nb_ss_ and intermetallics (silicides and others) can be rigid and/or creeping. Models [16] of the creep behavior of these alloys and comparison of the results of modelling with experimental data for alloys with about 37% volume fraction silicide, (i.e., with composites with weak matrix, see above) have shown that rigid Nb silicides have high creep exponent and poor creep. Chan [16] concluded (a) that creeping silicides with low creep exponent (n ≈ 1) are desirable, (b) that creeping or rigid phases with n ≥ 3 are undesirable, (c) that desirable phases for optimum creep should be rigid at low stresses and creep with low n at higher stresses and (d) that diffusional creep and Harper-Dorn creep in intermetallics are preferred over power law creep with n > 1. The creep of composites with strong (Nb_5_Si_3_) matrix has been simulated by Henshall et al. [17] using data for bulk unalloyed Nb_5_Si_3_ and Nb-1.25Si solid solution. This work under-predicted steady state creep rates and over-predicted primary creep strains compared with experimental data for Nb-10Si. 

**Figure 8:**
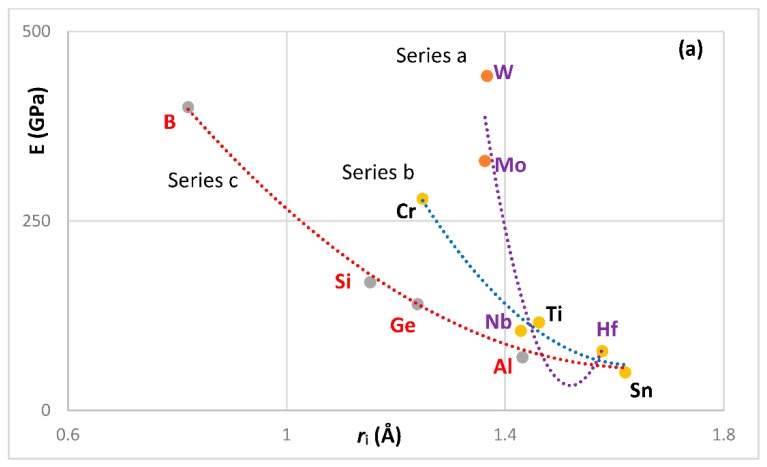

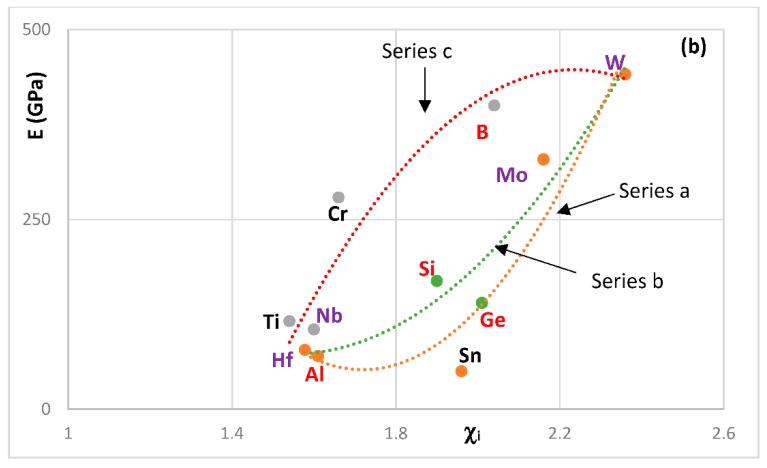
Young’s modulus (ordinate) E (GPa) of solute elements in Nb versus (abscissa) their atomic radius r_i_ (**a**) and Pauling electronegativity χ_i_ (**b**). In (**a**) elements in series a: Hf, Mo, Nb, Ti, W, in series b: Cr, Hf, Nb, Sn, Ti, in series c: Al, B, Ge, Nb, Si, Sn. In (**b**) elements in series a: Al, Hf, Mo, Sn, W, in series b: Al, Ge, Hf, Mo, W, in series c: Al, B, Cr, Hf, Nb, Ti, W.

Elastic constants depend on interatomic bonding and are important to understanding how the material will deform (see previous section). In reference [6] it was shown that the solutes in Nb belong in different groups when activation energy for diffusion (Q) and diffusivity (D) were plotted against atomic size or electronegativity. Figure 8 shows the Young’s (E) moduli of solute elements in Nb-silicide based alloys against atomic radius (Figure 8a) and electronegativity (Figure 8b). The solute elements fall in three groups with Boron in one of these groups (series c in both parts of Figure 8), consistent with the data for Nb-silicide based alloys in [46]. The transition metal (TM) and refractory metal (RM) elements in the series a, b and c in Figure 8 are in agreement with the ranking of substitutional solutes in binary Nb-X alloys in terms of their effect on creep strength [115].

### 2.3. Relationships between Alloy Parameters, Properties and Solute Concentrations

**Figure 9:**
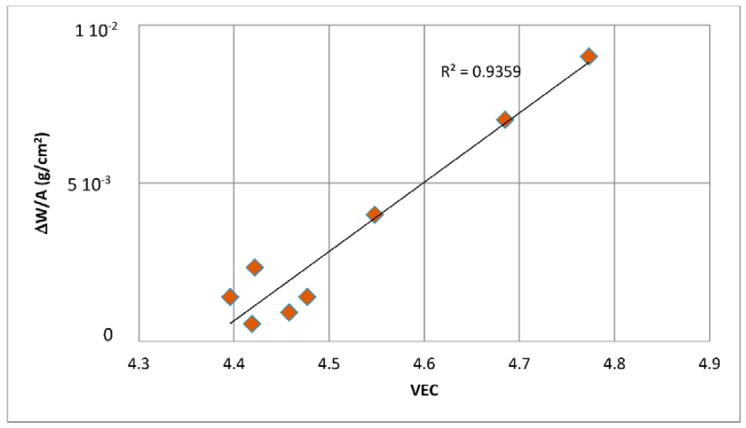
Weight-gain ΔW/A (g/cm^2^) (ordinate) at 800 °C versus (abscissa) the alloy parameter VEC for the elements Al, Cr, Ge, Hf, Mo, Nb, Si, Sn, Ta, Ti, W in Nb-silicide based alloys.

Correlations were found between weight gain per unit area (ΔW/A) after 100 h isothermal oxidation at 800 °C or 1200 °C and the alloy parameters Δχ, δ or VEC. Different functions (ΔW/A) = f_1_(Δχ), (ΔW/A) = f_2_(δ) or (ΔW/A) = f_3_(VEC) and plots like the one shown in Figure 9 were established for each temperature. In Figure 9 the R^2^ value for the linear fit of the data is 0.9359. In similar plots (not shown) of (ΔW/A) versus δ and (ΔW/A) versus Δχ the R^2^ values respectively were 0.932 and 0.949 for oxidation at 800 °C. For the oxidation at 1200 °C the R^2^ values for the (ΔW/A) versus Δχ, (ΔW/A) versus δ and (ΔW/A) versus VEC plots (not shown) were 0.935, 0.95 and 0.951, respectively. The oxidation data (isothermal weight gain) was from [14,15,59,109,116,117,118,119,120,121].

**Figure 10:**
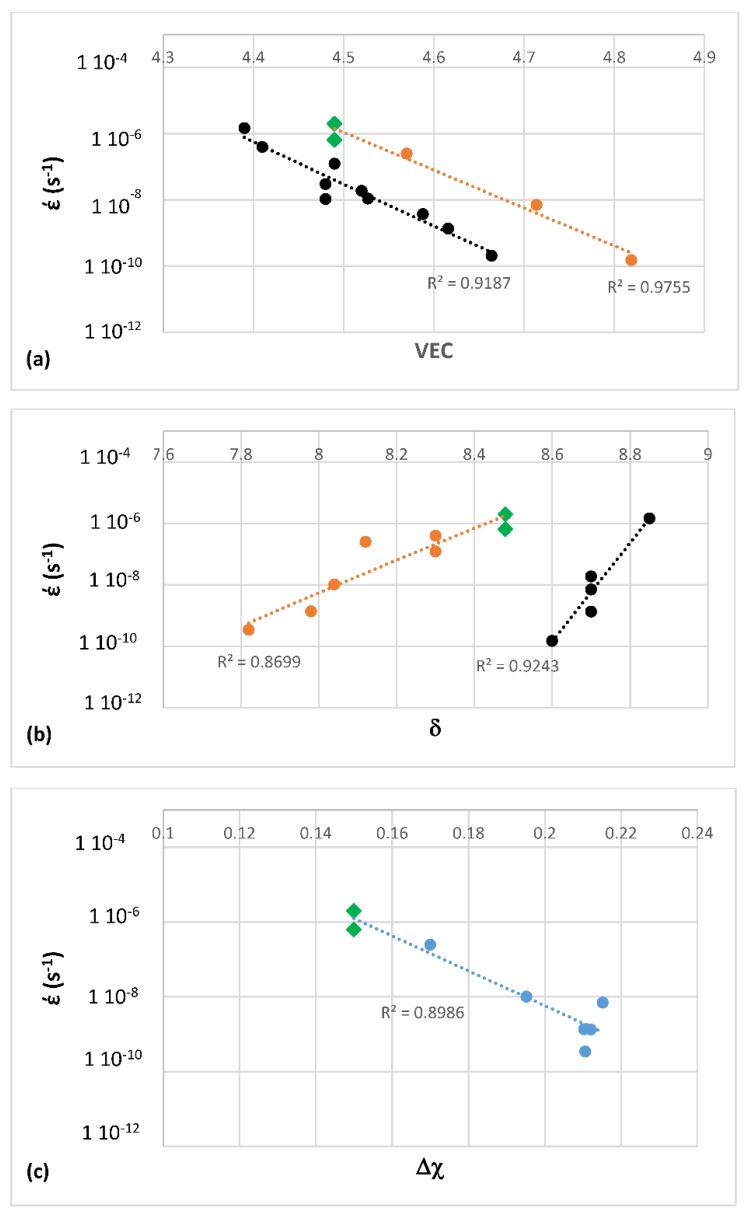
Creep rate (s^−1^) (ordinate) at 1050 °C and 100 MPa versus (abscissa) (**a**) VEC; (**b**) δ and (**c**) Δχ of Nb-silicide based alloys with Al, Cr, Hf, Mo, Si, Ti, W. In (**a**) the concentrations of Al, Si and Ti were different between the series a (R^2^ = 0.9755) and series b (R^2^ = 0.9187) alloys. In (**b**) the series a (R^2^ = 0.8699) and series b (R^2^ = 0.9243) alloys had different concentrations of Al and Ti. MASC alloy data is shown by green diamonds.

Figure 10 shows compressive creep data at 1050 °C and 100 MPa of the MASC alloy and other Nb-silicide based alloys [122]. The creep rate is plotted versus the parameters VEC, δ or Δχ. The creep rate decreases with increasing VEC or Δχ and increases with increasing δ. Creep rate of Nb-silicide based alloys at 1050 °C and 1200 °C for stresses higher than 100 and up to 300 MPa [122] also was found to be related to the alloy parameters δ, VEC and Δχ. The R^2^ value for the fit of data for creep rate at 1200 °C and 170 MPa was 0.9299 in έ = g_1_(δ) (figure not shown). The R^2^ value for the fit of data for creep rate at 1200 °C and 170 MPa in έ = g_2_(Δχ) and έ = g_3_(VEC) was 0.9586 and 0.9149, respectively (figures not shown). The size of the phases in the studied alloys was similar [7,9].

**Figure 11:**
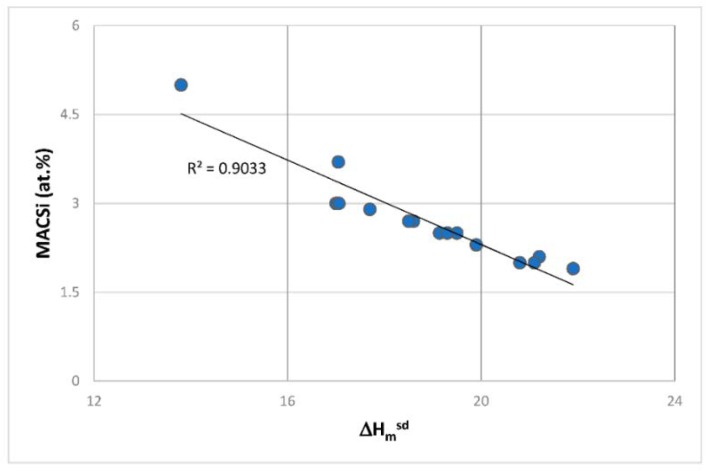
Macrosegregation of Si (MACSi, at.%) (ordinate) in Nb-silicide based alloys versus (abscissa) the ΔH_m_^sd^, see [12].

The macrosegregation of Si (MACSi) in cast Nb-silicide based alloys was discussed in [13]. Figure 11 shows such a relationship between MACSi = [C_max_^Si^ − C_min_^Si^] and ΔH_m_^sd^ with R^2^ = 0.9033. Similar relationships were found for the macrosegregation of Si versus ΔH_m_^sd^/ΔH_m_^sp^ or T_m_^sd^/T_m_^sp^ with R^2^ = 0.946 and R^2^ = 0.996, respectively (figures not shown). For the definition of the parameters ΔH_m_^sd^, ΔH_m_^sp^, T_m_^sd^ and T_m_^sp^ see [13].

**Figure 12:**
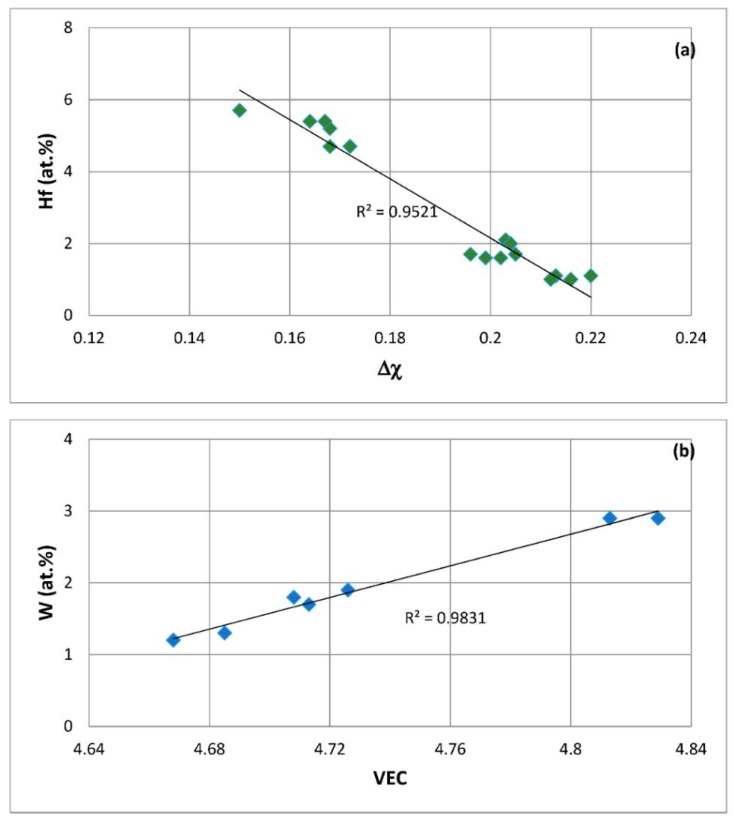
(**a**) (ordinate) Hf concentration (at.%) in alloy versus (abscissa) the alloy parameter Δχ for the elements Al, B, Cr, Ge, Hf, Mo, Nb, Si, Sn, Ta, Ti, W in Nb-silicide based alloys; (**b**) (ordinate) W concentration (at.%) in alloy versus (abscissa) the alloy parameter VEC for the elements Al, Cr, Ge, Hf, Mo, Nb, Si, Sn, Ti, W in Nb-silicide based alloys.

Having established relationships between properties and parameters of Nb-silicide based alloys, the next step was to find out whether the property goals and said relationships could be used to assist the design (selection) of Nb-silicide based alloys. Relationships between the alloy parameters and solute additions were sought. It was discovered that the concentrations of all solute elements in Nb-silicide based alloys can be expressed as functions of the alloy parameter Δχ. An example is shown in Figure 12a for the linear relationship Hf = ψ_1_(Δχ) for the concentration of Hf in Nb-silicide based alloys. The R^2^ value for the fit of the data in Figure 12a was 0.9521. Similar relationships were discovered for the solutes Al, Ge, Cr, Mo, Si, Sn, Ti, and W with R^2^ values 0.957, 0.926, 0.9513, 0.930, 0.916, 0.930, 0.915 and 0.973, respectively (figures not shown). The concentrations of solutes also could be expressed as functions of other alloy parameters; an example is shown in Figure 12b for W versus the alloy parameter VEC, where R^2^ = 0.9831.

**Figure 13:**
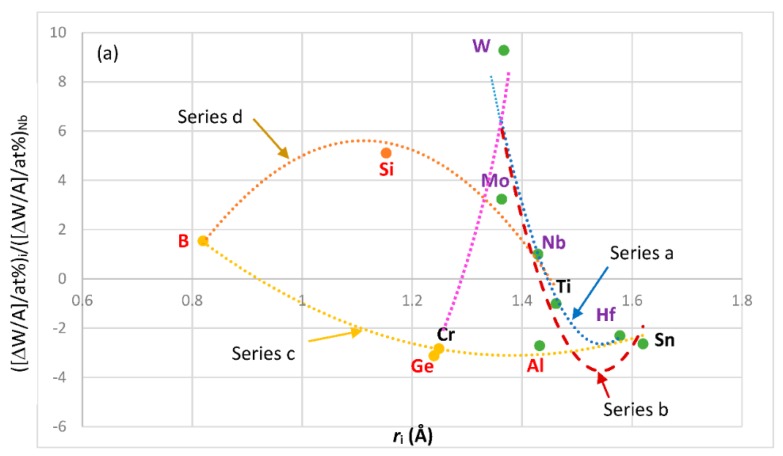

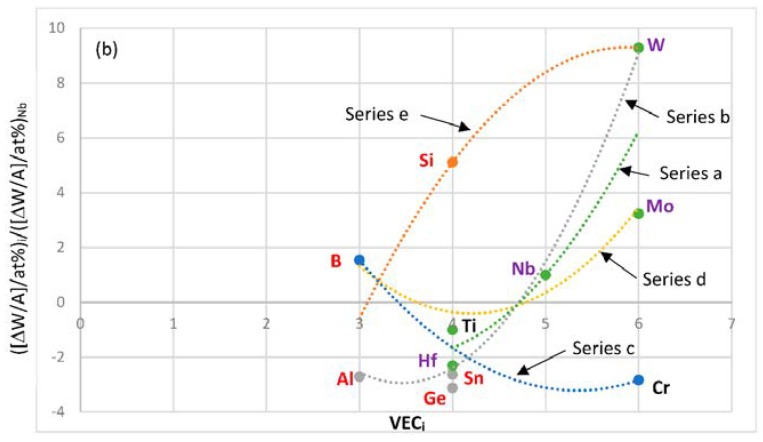
(Ordinate) ([ΔW/A]/at.%)_i_/([ΔW/A]/at.%)_Nb_ at 1200 °C versus (abscissa) atomic radius *r*_i_ (Å) (**a**) and VEC_i_ of element I (**b**). In (**a**) elements in series a: Hf, Mo, Nb, Ti, W, in series b: Al, Hf, Mo, Nb, Sn, Ti, W, in series c: Al, B, Cr, Ge, Hf, Sn, in series d: B, Mo, Nb, Si, Ti and in (**b**) elements in series a: Hf, Mo, Nb, Ti, W, in series b: Al, Ge, Hf, Nb, Sn, W, in series c: B, Cr, Hf, Ti, in series d: B, Mo, Nb, Ti and in series e: Al, B, Si, W.

The relationships between alloy weight gain in isothermal oxidation at 800 °C and 1200 °C and the alloy parameters VEC, δ and Δχ (for example, see Figure 9), and between the concentrations of elements in Nb-silicide based alloys and the alloy parameters were used to find out the contribution each element makes in weight gain in Nb-silicide based alloys at each temperature by expressing weight gain as a function of solute element concentration. The relevant equations were of the type [ΔW/A]_i_ (g/cm^2^) = a_i_ + b_i_C_i_ where i = Al, B, Cr, Ge, Hf, Mo, Nb, Si, Sn, Ti, W, and C is the concentration (at.%) of element i and a and b are constants. For example, for oxidation at 1200 °C the above constants for Al were a_Al_ = 6.22·10^−4^ g/cm^2^ and b_Al_ = 0.0105 g/cm^2^ at.% for Nb-silicide based alloys without Boron. The grouping of elements was based on the results in [46] and the available experimental data for isothermal oxidation. Figure 13 shows the contribution to weight gain of element i at 1200 °C normalized against the weight gain of Nb, versus atomic radius and VEC of element i. Positive values of ([ΔW/A]/at.%)_i_/([ΔW/A]/at.% )_Nb_ mean reduction in weight gain. The solutes with atomic size *r*_i_ < *r*_Nb_, namely B, Mo, Si and W, reduce weight gain, the solutes with atomic size *r*_i_ > *r*_Nb_, namely Al, Hf, Sn and Ti, increase weight gain. The solutes Cr and Ge, which have *r*_i_ < *r*_Nb_, fall in the same group as Mo and W but increase weight gain. It should be noted that the data in Figure 13 is only relevant to the concentrations of solute additions used in Nb-silicide based alloys. The solutes Al, B, Cr, Ge and Sn that are used to control the oxidation of Nb-silicide based alloys fall in the same group (series c in Figure 13a). Ti and Hf, which improve oxidation, belong in a separate group. The majority of the elements in series b, c and d in Figure 13b are the same as those in the same series in Figure 13a. Also, it should be noted that the same elements belong in the series a in the Figure 8a and Figure 13a,b and that there are similarities in the elements in series a, b and c in Figure 8b and Figure 13b.

The creep goal was given in the introduction. Experimental work has shown that the density of Nb-silicide based alloys can be around 7 g/cm^3^ [123] and that these alloys should have 16 < Si < 22 at.% [124] to meet this goal.

**Figure 14:**
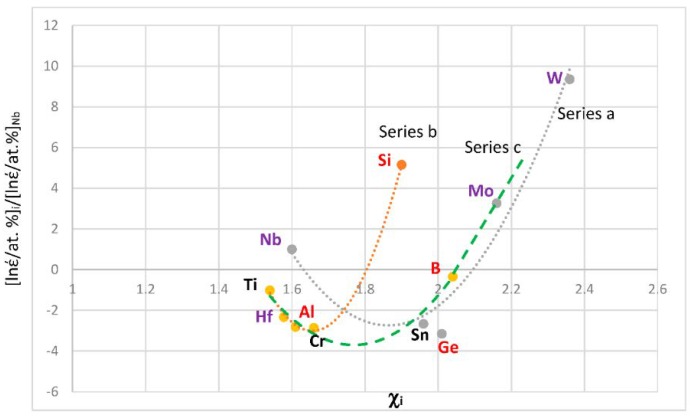
(Ordinate) [lnέ/at.%]_i_/[lnέ/at.%]_Nb_ at 1200 °C and 170 MPa (ordinate) versus (abscissa) Pauling electronegativity χ_i_ of element i. Elements in series a: B, Ge, Mo, Nb, Sn, W, in series b: Al, B, Hf, Si, Ti, in series c: Al, B, Cr, Hf, Mo, Sn, Ti, W.

The relationships between creep rate at 1200 °C and 170 MPa and the alloy parameters VEC, δ and Δχ and between the concentrations of elements in Nb-silicide based alloys and the alloy parameters were used to find out the contribution each element makes in creep rate for the creep goal conditions by expressing creep rate as a function of solute element concentration. The relevant equations were of the type lnέ = c_i_ + d_i_C_i_ where i = Al, B, Cr, Ge, Hf, Mo, Nb, Si, Sn, Ti, W, and C is the concentration (at.%) of element i and c and d are constants. For example, for Cr, c_Cr_ = −23.62 s^−1^ and d_Cr_ = 2.63 s^−1^ per at.%. Figure 14 shows the contribution to creep rate at 1200 °C and 170 MPa of element i normalized against the contribution to creep rate of Nb, versus Pauling electronegativity χ_i_ of element i. Positive values of [lnέ/at.%]_i_/[lnέ/at.%]_Nb_ mean decrease in creep rate. The elements Mo, Si and W contribute to decrease creep rate while Al, B, Cr, Ge, Hf, Sn and Ti increase creep rate. The ranking of RM alloying additions is in agreement with [115]. There are similarities in the elements in series c in the Figure 8b, Figure 13 and Figure 14, and in series c in Figure 8a and series a in Figure 14.

### 2.4. Approaches to Alloy Design and Selection

**Figure 15:**
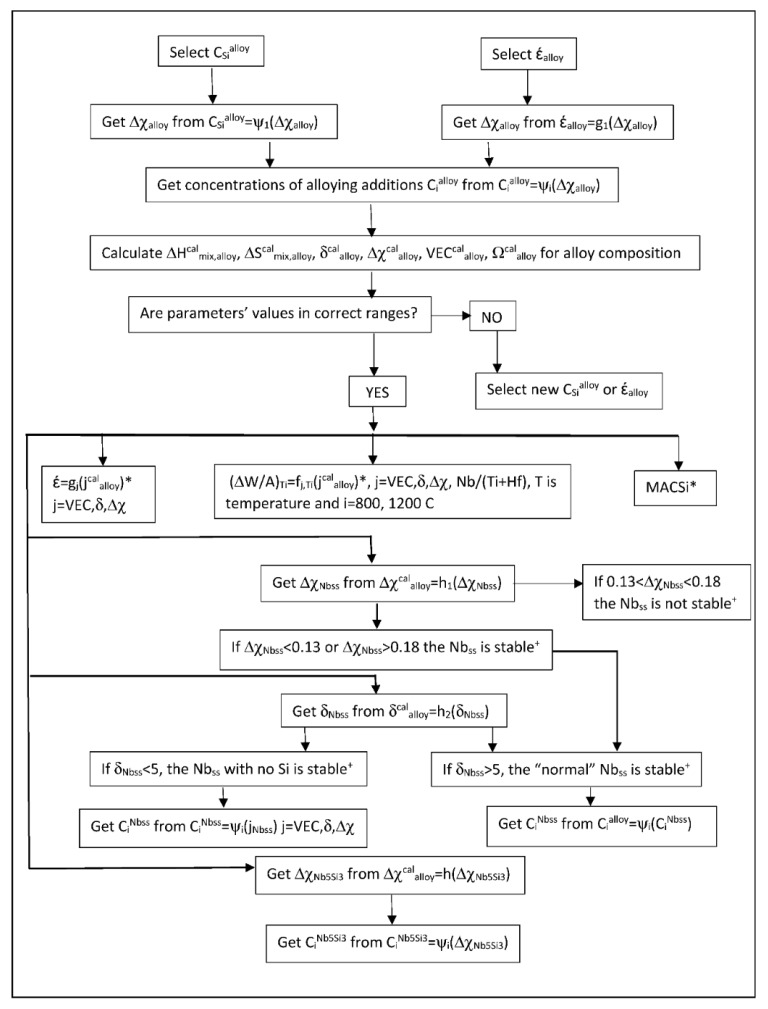
Schematic diagram of Niobium Intermetallic Composite Elaboration (NICE) alloy design(selection) methodology (* see text, + see [6]).

The final objective of the research was to use the above data to develop a design methodology. The first approach to design a Nb-silicide based alloy was based on selecting a Si concentration, for example this could be from the range of the concentration of Si for “best” creep (16 < Si < 22 at.% [124]), say 18 at.%. Next, this Si concentration was used to calculate the value of the parameter Δχ_alloy_ from the Si = ψ_1_(Δχ_alloy_) equation. The calculated value of Δχ_alloy_ was then used to calculate the concentrations of all other solute elements, for example Hf = ψ_1_(Δχ_alloy_) for Hf, see Figure 12a. The calculated concentrations of the solutes gave an alloy composition for which the values of the alloy parameters ΔH^cal^_mix,alloy_, ΔS^cal^_mix,alloy_, δ^cal^_alloy_, Δχ^cal^_alloy_, VEC^cal^_alloy_, Ω^cal^_alloy_ (sd/sp)^cal^ and {Nb/(Ti + Hf)}^cal^ were calculated as discussed in [46]. To proceed with the calculation (prediction) of properties (creep, macrosegregation and oxidation (weight gain after isothermal oxidation for 100 h at 800 °C and 1200 °C)) all the alloy parameters of the calculated alloy composition were checked to find out if they were in the ranges given in [46]. If the latter was the case, the creep rate at 1200 °C and 170 MPa was calculated using the function έ = g_2_(δ^cal^_alloy_) or έ = g_1_(Δχ^cal^_alloy_) or έ = g_3_(VEC^cal^_alloy_). The weight gain at 800 °C was calculated using the function ΔW/A = f_3_(VEC^cal^_alloy_), for example see Figure 9, or ΔW/A = f_2_(δ^cal^_alloy_) or ΔW/A = f_1_(Δχ^cal^_alloy_), and the weight gain at 1200 °C was calculated using similar functions for this temperature. The macrosegregation of Si in the cast alloy was predicted using relationships like that shown in Figure 11. The outlined procedure is shown in Figure 15.

An example of a Nb-silicide based alloy designed using the approach described above is the alloy Nb-21.93Ti-18Si-4.3Cr-4.06Al-4.9Hf-3.43Mo-1.03W-4.5Ge (at.%). We shall call this alloy A. This alloy has VEC^cal^_alloy_ = 4.513, δ^cal^_alloy_ = 8.79 and Δχ^cal^_alloy_ = 0.2. The parameters of this alloy are in the ranges for Nb-silicide based alloys [46]. The calculated (predicted) weight gains at 800 °C and 1200 °C, respectively are 8.9 mg/cm^2^ and 64.5 mg/cm^2^ and the macrosegregation of Si is 5.5 at.%. The predicted creep rate at 1200 °C and 170 MPa is lower than the creep goal (see below). The weight gain after 100 h at 1200 °C is significantly higher than that of the single crystal Ni based superalloy CMSX-4 (1 to 4 mg/cm^2^).

Another approach was to use έ = g_1_(Δχ_alloy_) and a selected value of the creep rate, for example use the creep goal (see Section 2.3) to set έ, and then to solve the equation for Δχ_alloy_ and then use the latter to calculate the concentrations of the solutes and thus the alloy composition as described above, see Figure 15.

An alternative approach to alloy design (selection) could be to use the dependence of weight gain at 800 °C and 1200 °C on the alloy parameters VEC and Δχ (i.e., use the functions [ΔW/A]_3_ = f_3_(VEC) and [ΔW/A]_1_ = f_1_(Δχ)) and the fact that the concentration of Si in the alloy is related to each of these two parameters with functions Si = ψ_1_(Δχ) and Si = ψ_3_(VEC). The weight gain functions can thus be re-written as functions of Si concentration in the alloy, and from [ΔW/A]_1_ = [ΔW/A]_3_ the Si concentration can be calculated at each temperature. The Si concentration in the alloy is then taken as the average of the two concentrations calculated for 800 °C and 1200 °C (the difference between the two calculated Si concentrations is ≤1 at.%). The Si concentration is accepted if it is in the range required for “best” creep (16 < Si < 22 at.% [124]). The accepted Si concentration is then used to get Δχ_alloy_ and the latter to calculate the concentrations of the other solute elements, following the same procedure as described previously (see Figure 15). An example of an Nb-silicide based alloy designed using this approach is the alloy Nb-19.3Ti-18.5Si-4.3Mo-3.8Hf-3.8Sn-3.7Ge-3.3Cr-3.1Al-1.3W (at.%). We shall call this alloy B. This alloy has VEC^cal^_alloy_ = 4.536, δ^cal^_alloy_ = 9.4 and Δχ^cal^_alloy_ = 0.2086. The parameters of this alloy are in the ranges for Nb-silicide based alloys [46]. The calculated (predicted) weight gains for isothermal oxidation for 100 h at 800 °C and 1200 °C, respectively are 5 mg/cm^2^ and 39 mg/cm^2^ and the macrosegregation of Si is 5.1 at.%. The calculated creep rate at 1200 °C and 170 MPa does not meet the creep goal (see below).

Another approach to alloy design was to use the relationship of creep rate with the alloy parameter Δχ and write έ = g_1_(Δχ) as a function of weight gains at 800 °C and 1200 °C given that [ΔW/A]_1,i_ = f_1,i_(Δχ) (i = 800, 1200) and then calculate the weight gains at 800 °C and 1200 °C for the creep goal. Next, from the weight gain equation, the value of Δχ_alloy_ is calculated, and the latter is subsequently used to calculate the concentrations of all solute elements, as described previously (see Figure 11).

Given that the weight gain for oxidation at a particular temperature is related to more than one of the studied parameters, as a first approximation, the calculated alloy weight gain at 800 °C or 1200 °C was taken to be the average of the values calculated using each of the three alloy parameters δ, Δχ and VEC. The weight gains calculated using each parameter at a given temperature were not significantly different from each other. The same approach was used for macrosegregation, meaning the macrosegregation of Si was the average of the values calculated using each of the parameters discussed above and in [13].

In the case of creep there can be at least an order of magnitude difference between the values calculated from the relationships with atomic size, electronegativity and valence electron concentration (VEC). For alloys that do not oxidize catastrophically in pest oxidation and at high temperatures the creep rate calculated from the relationship with VEC is higher than 10^−7^ s^−1^ and the relationships between the atomic size or electronegativity with alloy creep rate give the latter respectively lower than 10^−6^ and 10^−7^ s^−1^. This is attributed (a) to different contributions to creep from each parameter given that electronegativity links with all the solutes in Nb-silicide based alloys, the atomic size describes the alloying behavior of the Nb_ss_, C14-NbCr_2_ Laves and A15-Nb_3_X phases, and electronegativity and VEC describe the alloying behavior of all phases in Nb-silicide based alloys [6,41,46,47,48], (b) to the different contributions of elements to creep (Figure 7B, Figure 8b and Figure 14), (c) to the anticipated changes in the properties (i) of Nb_5_Si_3_ silicides because of the shift of Nb_5_Si_3_ silicides in the Δχ versus VEC maps depending on solute elements substituting Nb and Si in the silicide (see [47] and Figure 7B), and (ii) of the Nb_ss_ because of changes in the composition of the solid solution (Figure 6 and Figure 7C) and (d) to changes of the elastic properties of the Nb_5_Si_3_ silicides with alloying [104]. 

Not the same values of the parameters δ, Δχ and VEC are calculated if one were to use the creep goal and some isothermal oxidation (weight gain) targets, say 1 mg/cm^2^ and 20 mg/cm^2^, respectively for 800 °C and 1200 °C. (Note that these targets are not the same as the oxidation goal. The latter was given as “a recession rate of less than 0.25 μm/hour at 1315 °C” [1]. This goal was derived from the requirement of achieving the oxidation life at 1315 °C that 2nd generation single crystal Ni-based superalloys presently exhibit at 1150 °C). The former oxidation target is achieved in Nb-silicide based alloys that do not pest and the latter is considered realistic and has been achieved. The alloy VEC values decrease from the creep goal to weight gain goal for oxidation at 800 and at 1200 °C, and the opposite is the case for the values of the alloy parameter δ. The alloy parameter Δχ has the highest value for the oxidation weight gain target at 800 °C and the lowest for the creep goal. This means that for the current property goals it is unlikely that an alloy could be designed where both creep and oxidation property goals can be met simultaneously. However, it is possible (i) to design (select) alloys where one of the property goals is surpassed and the other is met very closely or (ii) to design alloys where both goals are met closely. Both (i) and (ii) have been achieved in the author’s research group. It is the opinion of the author that attaining a weight gain of 1 to 4 mg/cm^2^, which is the weight gained by the single crystal Ni based superalloy CMSX-4 alloy after 100 h isothermal oxidation in air at 1200 °C, in polycrystalline Nb-silicide based alloys at 1200 °C is unrealistic unless the vol % of Nb_ss_ is extremely low in the latter.

In the alloy design (selection) methodology outlined in this paper, the criterion used to select an alloy for further study considering the predicted creep rate at the creep goal conditions is the following. “If the calculated creep rates from the relationships based on atomic size, electronegativity and VEC are higher than 10^−7^ s^−1^ the alloy is considered highly unlikely to meet the creep goal”, which is “the creep strength should be greater than 170 MPa at a creep rate of 2 10^−^^8^ s^−^^1^ at 1200 °C” [1]. Using this criterion, the alloys A and B (see above) were predicted not to meet the creep goal.

Each approach to alloy design (selection) that has been discussed above would give an alloy composition for which solidification path, volume fractions of phases and phase equilibria at different temperatures could be calculated, provided thermodynamic data was available. This task is not possible currently, for the reasons discussed in the introduction. Using knowledge gained from on-going research it is possible to make some informed predictions for the selected alloys. The latter and the calculated (predicted) properties can be tested experimentally. For example, the alloy A should have βNb_5_Si_3_ as its primary phase, in its microstructure tetragonal Nb_5_Si_3_ and Nb_ss_ should be stable at 1500 °C but not the C14 NbCr_2_ Laves and A15 intermetallic phases and the alloy should not pest. The alloy B should have βNb_5_Si_3_ as its primary phase, eutectic with Nb_ss_ and Nb_5_Si_3_ should form (see below), in its microstructure tetragonal Nb_5_Si_3_, Nb_ss_ (most likely solid solution with no Si) and A15 intermetallic should be stable at 1500 °C but not C14 NbCr_2_ Laves phase and the alloy should not pest.

**Figure 16:**
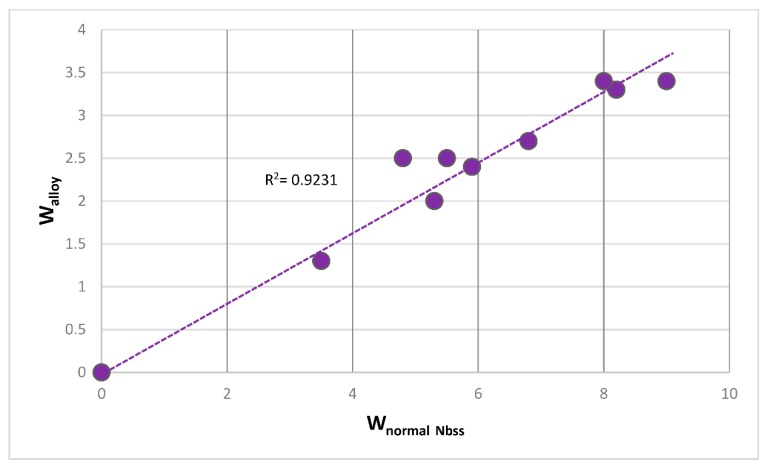
W in alloy (ordinate) versus (abscissa) W in normal Nb_ss_. Data for Nb-silicide based alloys with Al, Cr, Ge, Hf, Mo, Nb, Si, Sn, Ta, Ti, W.

**Figure 17:**
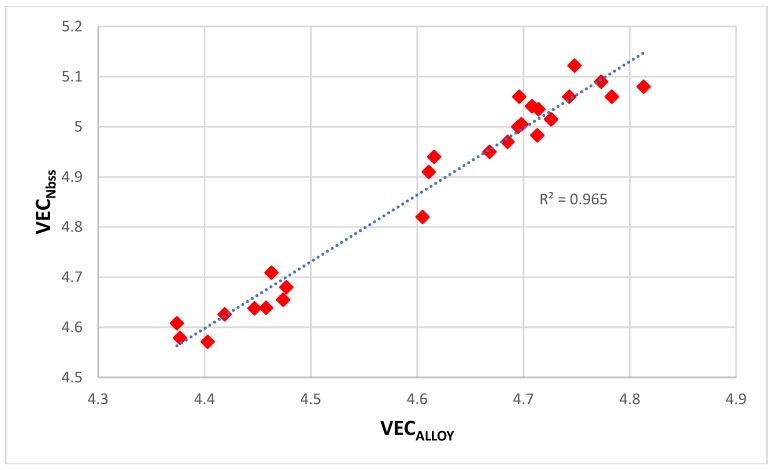
Relationship between VEC_Nbss_ and VEC_alloy_. Data for Nb-silicide based alloys with Al, Cr, Ge, Hf, Mo, Si, Sn, Ta, Ti, W.

The parameters VEC, Δχ and δ also are important for the types of bcc Nb_ss_ that form in Nb-silicide based alloys, see [6]. It is known that the solubility of Cr in bcc Nb_ss_ depends on that of Ti in the solid solution [9,40]. Relationships between solute elements in the alloy and solid solution exist, for example see Figure 16 for the relationship (R^2^ = 0.9231) between W in the alloy and W in the normal Nb_ss_. There are also relationships between Δχ_alloy_ and Δχ_Nbss_, δ_alloy_ and δ_Nbss_ and VEC_alloy_ and VEC_Nbss_, for example see Figure 17 for the relationship between VEC_alloy_ and VEC_Nbss_ (R^2^ = 0.965) Such relationships make it possible to calculate (predict) the composition of the solid solution in a designed (selected) alloy, see Figure 15. There are also relationships between the parameters of the alloy and Nb_5_Si_3_, for example Δχ_alloy_ = h(Δχ_Nb5Si3_) with R^2^ = 0.9081 for alloying elements Al, B, Cr, Ge, Hf, Si, Sn, Ta, Ti, from which the Δχ_Nb5Si3_ is calculated and the latter is then used to get concentrations of solute additions in the silicide from, say C_i_^Nb5Si3^= ψ_i_(Δχ_Nb5Si3_), see Figure 15.

**Figure 18:**
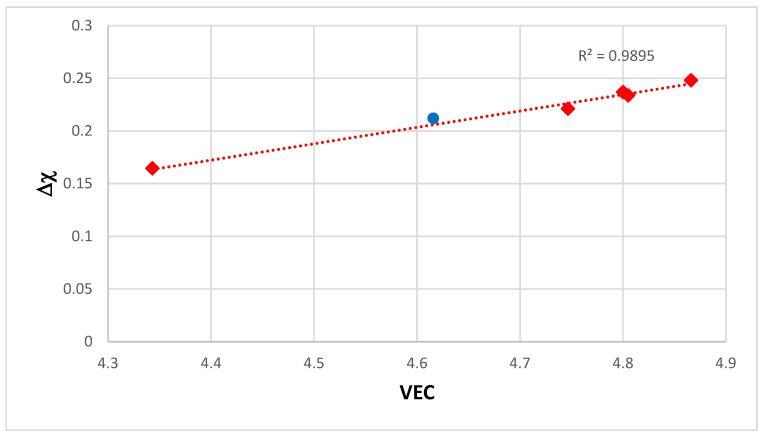
(Ordinate) Δχ versus (abscissa) VEC of eutectics with Nb_ss_ and Nb_5_Si_3_. Data for series e (R^2^ = 0.9895) in Figure 5 in reference. [41], alloying elements Al, Cr, Ge, Hf, Mo, Si, Sn, Ti, W. The blue color data point corresponds to alloy B (see text).

It is possible to predict the presence or not of a eutectic with Nb_ss_ and Nb_5_Si_3_ and whether it will be a rich or poor in Ti eutectic in a Nb-silicide based alloy designed using the alloy design methodology described in this paper. The parameters Δχ and VEC of the eutectics with Nb_ss_ and Nb_5_Si_3_ [41] can account for their alloying behavior and are related to the parameters Δχ and VEC of the Nb-silicide based alloys with linear relationships with R^2^ = 0.9325 and R^2^ = 0.9517, respectively (figures not shown). For an alloy selected using the methodology described in this paper, the Δχ_eutectic_ and VEC_eutectic_ will be calculated from the aforementioned relationships and Figure 5 in reference [41] could be used to find out whether a eutectic with Nb_ss_ and Nb_5_Si_3_ would form. Then Figure 4 in reference [41] would be used to predict whether the eutectic will be rich or poor in Ti. The relevant part of Figure 5 in reference [41] is reproduced in Figure 18 in the present paper. The alloy B will be used to demonstrate the above procedure. The composition of the alloy B is Nb-19.3Ti-18.5Si-4.3Mo-3.8Hf-3.8Sn-3.7Ge-3.3Cr-3.1Al-1.3W (at.%), see above. The calculated parameters for a eutectic with Nb_ss_ and Nb_5_Si_3_ in alloy B are VEC_eutectic_ = 4.602 and Δχ_eutectic_ = 0.2156. Figure 18 shows that a eutectic with Nb_ss_ and Nb_5_Si_3_ can form in this alloy (the data point is very close to the line for eutectics with the same elements as those in the alloy B (series e in Figure 5 in reference [41]). Figure 4 in reference [41] shows that in the alloy B a eutectic with Nb_ss_ and Nb_5_Si_3_ and the above Δχ_eutectic_ and VEC_eutectic_ values would be poor in Ti. Thus, the alloy B is predicted to have a poor in Ti eutectic with Nb_ss_ and Nb_5_Si_3_.

### 2.5. Other Relationships

**Figure 19:**
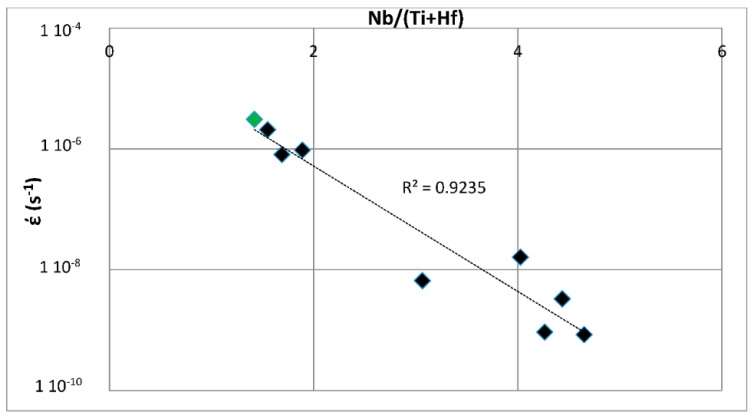
Creep rate (s^−1^) at 1050 °C and 150 MPa (ordinate) versus (abscissa) Nb/(Ti + Hf) ratio. Elements in Nb silicide-based alloys Al, Cr, Hf, Mo, Nb, Si, Ti, W. The MASC alloy is indicated in green. For MASC see [1,2]. Creep data from the EU FP6-ULTMAT project [122].

The type of Nb_5_Si_3_ silicide (meaning tetragonal α and β Nb_5_Si_3_ and hexagonal γNb_5_Si_3_) that is stable in the microstructure of Nb-silicide based alloys depends on all the solute additions in the alloy and the actual concentrations of Ti and Hf in the alloy and silicide, and is critical for the creep properties of the silicide and alloy [1,2,47,111]. For example, with the addition(s) of Cr, Mo, Ta and W often the βNb_5_Si_3_ is stabilized and with the additions of Hf, Ti and Zr the hexagonal Nb_5_Si_3_ silicide can be stabilized depending on the concentration of these elements in the alloy and silicide. Hexagonal Nb_5_Si_3_ silicide is undesirable for creep [1,124]. Titanium and Hf additions also are important for oxidation. The Nb/(Ti + Hf) ratio is often used as an indicator of the structure (tetragonal or hexagonal) of the Nb_5_Si_3_ silicide. If the value of this ratio in the alloy and Nb_5_Si_3_ is less than 1 it is likely that the latter has hexagonal structure and the likelihood of the latter increases the smaller this ratio becomes. Figure 19 shows that the creep rate decreases with increasing Nb/(Ti + Hf) ratio, i.e., when tetragonal Nb_5_Si_3_ silicide(s) is(are) present in the microstructure. The MASC alloy is included in the Figure 19.

**Figure 20:**
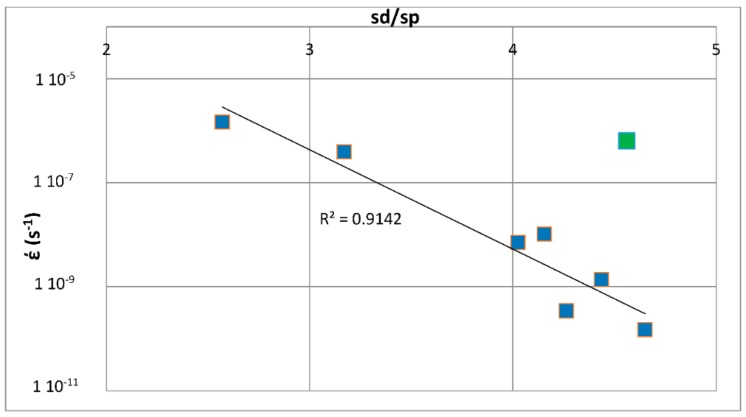
Creep rate (s^−1^) at 1050 °C and 100 MPa (ordinate) versus (abscissa) sd/sp ratio. Elements in Nb silicide-based alloys Al, Cr, Hf, Mo, Nb, Si, Ti, W. The MASC alloy is indicated in green. For MASC see [1,2]. Creep data from the EU FP6-ULTMAT project [122].

The Nb/(Ti + Hf) ratio considers only Nb and two sd elements that promote hexagonal Nb_5_Si_3_ silicide. In Nb-silicide based alloys, however, there are other TM and RM elements and simple metal and metalloid elements and Si, all of which contribute to establish, for given processing route(s), microstructure(s) with specific creep and oxidation properties (and macrosegregation of Si in the cast alloy). There exist relationships between the aforementioned and the alloy parameters VEC, Δχ and δ. A parameter that considers all the elements in the alloy is the sd/sp ratio [46]. The latter can be related to creep, like the Nb/(Ti + Hf) ratio. Figure 20 shows such a relationship for alloys with the elements indicated in the figure caption. The MASC alloy is included in this figure to demonstrate that the sd/sp ratio alone is not sufficient for alloy design (selection) and that this ratio should be used with great care.

## 3. Summary

The current situation vis-à-vis composition-process-microstructure-property relationships and thermodynamic data for Nb-silicide based alloys is unsatisfactory and makes it impossible to design (select) new alloys using methodologies that have been established for other alloy families. There are relationships between the alloy parameters δ, Δχ and VEC, and the concentrations of solute elements in Nb-silicide based alloys and their creep and oxidation (weight gain) properties. Such relationships were demonstrated in the paper and different approaches to design (select) new alloys using them and property goals for Nb-silicide based alloys were discussed.
